# Nd Recovery from Wastewater with Magnetic Calcium Alginate ((1,4)-β-d-Mannuronic Acid and α-L-Guluronic Acid) Hydrogels

**DOI:** 10.1021/acsomega.2c08221

**Published:** 2023-05-03

**Authors:** Emircan Uysal, Elif Emil-Kaya, Duygu Yesiltepe-Ozcelik, Sebahattin Gurmen

**Affiliations:** † Department of Metallurgical and Materials Engineering, Istanbul Technical University, 34469 Istanbul, Türkiye; ‡ IME Process Metallurgy and Metal Recycling, RWTH Aachen University, Aachen, Nodrhein-Westfalen DE 52062, Germany

## Abstract

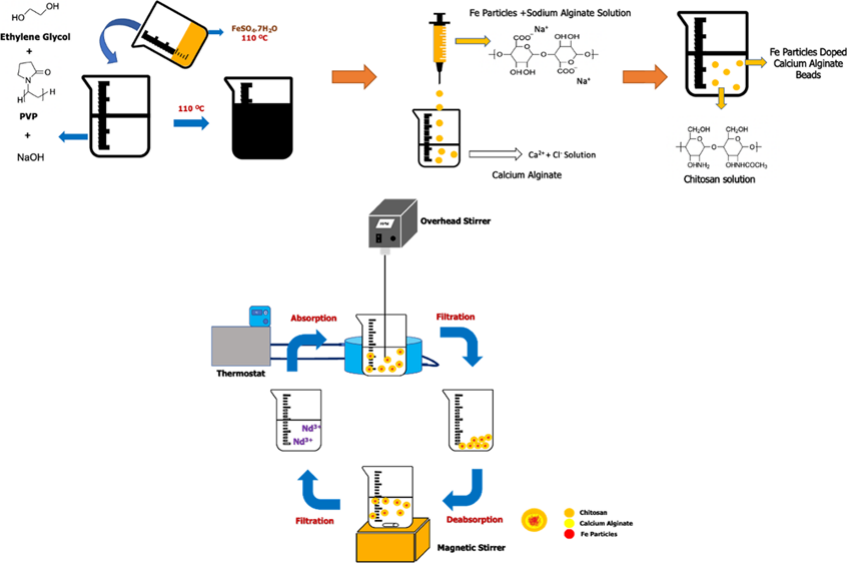

In this study, a magnetic adsorbent material was produced, by environmentally friendly and inexpensive precursor materials, to clean wastewater that may result from primary and secondary rare earth metal (REM) production. Then, the absorption of Nd^3+^ ions from wastewater was done and this process’s kinetic and isotherm models were developed. Thus, the removal of Nd^3+^ from wastewater with magnetic materials was accomplished, and then, this precious metal was recovered by using different acid media. First, Fe sub-micron particles were successfully produced by the polyol method. To increase the stability of Fe-based particles, their surfaces were covered with an oxide layer, and the average thickness was determined as 16 nm. The synthesized Fe particles were added into the calcium alginate beads and then coated with chitosan to increase the pH stability of the gels. The chemical composition of the gels was determined by Fourier transform infrared spectroscopy, the thermal properties were determined by differential scanning calorimetry, and the magnetic properties were determined by vibrating-sample magnetometer analysis. The magnetic saturation of the hydrogels was 0.297 emu/g. After the production of magnetic calcium alginate hydrogels, Nd^3+^ ion removal from wastewater was done. Wastewater was cleaned with 94.22% efficiency. The kinetic models of the adsorption study were derived, and isotherm studies were done. Adsorption reaction fitted different kinetic models at different time intervals and the Freundlich isotherm model. The effect of pH, temperature, and solid–liquid ratio on the system was determined and the thermodynamic constants of the system were calculated. After the adsorption studies, Nd^3+^ ions were regenerated in different acid environments and achieved an 87.48% efficiency value. The removal of Nd^3+^ ions from wastewater was carried out with high efficiency, the gels obtained as a result of adsorption were regenerated with high efficiency by using acid media, and it was predicted that the gels could be reused. This study is thought to have reference results not only for the removal of REM from wastewater by magnetic adsorption materials but also for the adsorption of heavy metals from wastewater.

## 1 Introduction

Today, most countries are placing unusual pushes on water resources. The global population is growing fast, and guesses demonstrate that with current implementations, the world will face a 40% deficiency between forecast demand and accessible supply of water by 2030. In addition, chronic water scarcity, hydrological changeability, and extreme weather events (such as droughts and floods) are noticed as some of the biggest risks to global prosperity and stability. Acceptance of the role that water scarcity and drought are playing in aggravating fragility and conflict is increasing.^1,2^ The most important issue that requires immediate attention in today’s society is heavy metal ion pollution, which is also one of the major problems.^3^ To find powerful ways to purify water at lower costs and with less energy, while also limiting the overall environmental effect, addressing these difficulties has spurred a significant amount of research. The most common techniques for removing heavy metal ions from water pollution are distillation, catalysis, electrochemical precipitation, solvent extraction, crystallization, oxidation, ion exchange, membrane separation, and adsorption techniques; among these, the adsorption technique is thought to be the simplest and most efficient due to its high efficiency, reproducibility, and adaptable material design.^3−6^ The use of magnetic adsorbent materials in the adsorption processes has become an increasingly attractive issue. Some authors have reported that heavy metals can be removed from wastewater by using magnetic nanoparticles.^7−12^ In this study, magnetic Fe-based sub-micron particles were produced and used. Fe-based particles were produced by the polyol method, which is an environmentally friendly, low cost, and easy process. The polyol method involves suspending the metal precursor in a polyol solvent with more than two hydroxyl groups and afterward heating the solution to a refluxing temperature. This method has been used to synthesize metallic, oxide, and semiconductor NPs.^13,14^ It is possible to reduce many metals in polyol media, from metal complexes.^15^ Reduction of Fe metal was found to be more difficult compared to noble metals due to its electro-reduction potential. The reduction mechanism of Co and Ni metals in the polyol environment has been previously reported by some authors.^16−18^ Acetaldehyde and diacetyl were found as byproducts in some studies, but it was observed that the presence of [OH]^−^ ions in the system provided acetaldehyde formation and metal reduction.^16^ However, Takashi et al. claimed that no acetaldehyde was formed during the reduction of Co with EG and they predicted that the diacetyl molecules formed could result from the use of catalysts such as Pd that could increase the degradation of organic structures.^17^ Shengming et al. calculated the EG-Ni-H_2_O pourbaix diagram thermodynamically in the study of metallic Ni reduction from Ni(NO_3_)_2_ with ethylene glycol; they claimed that diacetylene is stable at low pH and stated that oxidation of diacetylene to H_2_CO_3_, HCO_3_^–^, and CO_3_^2–^ structures occur at high pH; also, they stated that by adding CaCl_2_ to the system, CaCO_3_ precipitated.^18^ The CaCO_3_ precipitation reaction they gave is given in eq 1.^18^
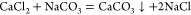
1

In this study, Na_2_CO_3_ formation was examined with the energy dispersive spectroscopy (EDS) results and discussed in the following sections. In general, the reduction has occurred in all studies, and different ideas have been advocated about its mechanism. The agreed point is that the coordination shell of the ions is changed with the addition of sodium hydroxide so that reduction is possible in the polyol medium with a basic medium, and reduction is carried out. [OH]^−^ ions increase the potential to reduce ionic species with the polyol, and the reaction rate is a function of the reduction potential of polyol, the concentration of Fe and [OH]^−^ ions, and also temperature.^19^ Joseyphus et al. showed that the reduction reaction with ethylene glycol from the FeCl_2_·4H_2_O precursor; [OH^–^]/Fe ion ratio has to be at a critical ratio, and it is important for reduction.^19^ Furthermore, in studies on Fe reduction, the formation of Fe oxide phases has been observed; Fe_3_O_4_ and γ-Fe_2_O_3_ occurred.^19,20^

Furthermore, in adsorption studies by doping metallic nanoparticles into different polymeric structures, various advantages such as increasing the pH stability of the particles and facilitating the regeneration of metal ions are provided. Recovery of heavy metal ions from wastewater by doping magnetic particles into the hydrogel matrix has been reported by some authors.^21,22^ Hydrogels are three-dimensional, cross-linked hydrophilic polymer chains that can absorb and keep in significant amounts of water, without dissolving or losing their three-dimensional structures.^23,24^ Hydrogels can be designed with controllable responses to shrink or expand with changes in external environmental conditions. They may perform remarkable volume transitions in response to a diversity of physical and chemical stimuli.^25^ Hydrogels are classified into two categories: chemical gels that have covalently bonded cross-linked chains and physical gels whose chains are kept together by molecular entanglements and/or secondary forces including ionic, hydrogen bonding, or hydrophobic interactions.^23,25^ One of the most important precursor materials of hydrogel materials is alginates. Alginate is a naturally occurring polysaccharide that includes homopolymeric (1,4)-d-mannuronic acid (M) and (1,4-l-guluronic acid (G) blocks coupled with different sequences or blocks.^12^ It is nontoxic, inexpensive, and possesses gelling capabilities because of ionic interactions with divalent cations.^12^ Moreover, it is a biocompatible and biodegradable material that can be used in areas where toxicity is most important, such as the food industry.^26^ It forms rigid hydrogels by interacting physically with carboxyl groups in the alginate structure and divalent or multivalent metal ions, forming the structure called “Egg-Box”. In this study, alginate hydrogels were used because they are both inexpensive and environmentally friendly. However, it is known that the carboxyl group of alginate becomes protonated and loses its stability in low-pH environments such as wastewater. For this reason, alginate hydrogels need to be coated with a second polymer to increase their stability at low pH. The pH stability of the gels was increased by coating them with chitosan. Chitosan, the fully or partially deacetylated form of chitin, is a common and abundant polymer in nature as the basic supporting structure for many living organisms, including fungi, crustaceans, insects, and arthropods.^27^ Chitosan is obtained by the deacetylation of chitin. It contains deacetylated and un-deacetylated groups in chitosan. At low pH, the deacetylated group is protonated and becomes water-soluble. The positive charge of this group and the negative charge of the alginate provide an electronegative coating of the alginate.

In this study, REM was tried to be removed from wastewater, and adsorbent REMs were tried to be recovered with different acidic media. First, sub-micron Fe-based particles were produced by the polyol method, the produced particles were doped into calcium alginate hydrogels, and the stability of the produced Fe particle-doped hydrogels was increased by coating with chitosan. The magnetic saturation of the produced material has been kept low by considering the regeneration studies. For this reason, the effects of temperature, pH, and absorbant-wastewater ratio parameters in the process of Nd^3+^ absorption from wastewater were examined and the optimization of the parameters was studied. The Fe-based particles were characterized by X-ray diffraction (XRD), scanning electron microscopy (SEM) and energy dispersive spectroscopy (EDS), differential scanning calorimetry (DSC), and Fourier transform infrared (FT-IR) spectroscopy. The obtained hydrogels were characterized by FT-IR and DSC, and the magnetic properties were determined by vibrating-sample magnetometer (VSM) analysis. The crystallite size of the synthesized Fe particles was calculated by modified Debye–Scherrer (MDS) and Williamson–Hall analysis (W–H) based on the uniform deformation model (UDM). Adsorption kinetic models, which include pseudo-first-order, pseudo-second-order, Weber-Morris and Elovich, adsorption isotherms, which are Langmuir and Freundlich, thermodynamical constants and different parameters, which are time, temperature, pH, and solid to liquid ratio, effects on the adsorption process were evaluated. This work may provide a new perspective on Fe-chitosan material synthesis and also rare earth metals (REMs) from wastewater and removal.

## 2 Materials and Methods

### 2.1 Materials

FeSO_4_·7H_2_O (TEKKİM, Türkiye) was used as a precursor in the synthesis of Fe particles, polyvinylpyrrolidone (PVP, *m*_w_ = 40,000 mol/g, Sigma Aldrich, USA) as the surface active material, and ethylene glycol (Merck, Germany) and NaOH (TEKKİM, Türkiye) were used. Sodium alginate (ISOLAB, Germany), as the precursor powder, and calcium chloride (E–401, Kimbiotek Kimyevi Maddeler San. Tic. A.Ş., Türkiye), as the crosslinking agent, were used in gel synthesis. Chitosan (Sigma Aldrich, Switzerland) solution was prepared with 2% acetic acid (Merck, Germany) solution and gels were coated with chitosan. The wastewater used in the adsorption experiments was prepared by using the starting material Nd_2_(SO_4_)_3_ (Merck, USA). The pH of the wastewater was adjusted using 0.1 M HCl (37%, Merck, Germany) and 0.1 M NaOH (TEKKİM, Turkey) solutions. In deadsorption studies, 0.1 M H_2_SO_4_ (95–98%, TEKKİM, Turkey), HCl (37%, Merck, Germany), HNO_3_ (65%, TEKKİM, Türkiye), L (+)-ascorbic acid (Merck, China), glycolic acid (Sigma Aldrich, USA), and L(+)-tartaric acid (Merck, Germany) were used. Ethanol (Merck, Germany), acetone (99.5%, Tekkim, Türkiye), and de-ionized water (produced from Milli-q, ITU, Türkiye) were used at different stages.

### 2.2 Synthesis of Fe Particles

The reduction of metallic Fe particles in the polyol medium was achieved by modifying the method described in another study.^19^ Two different solutions were prepared, homogenized, and heated to 110 °C, separately. The first solution contains 20 mL of ethylene glycol, 20 mL of deionized water, 0.8 g of NaOH, and 0.6 g of PVP, while the second solution contains 10 mL of deionized water and 0.44 g of FeSO_4_·7H_2_O. After mixing the two solutions, the reduction was carried out at 110 °C for 30 min. After the reduction is complete, the solution is rapidly reduced to 5 °C (model: GPWB-240 V PolyScience) to prevent agglomeration. After the particles were washed with acetone, deionized water, and ethanol, produced Fe-based particles were centrifuged (model: M19 Electromag) at 4000 rpm for 30 min.

### 2.3 Producing Chitosan Coated, Fe-Doped Calcium Alginate Hydrogel

Sodium alginate powders (2 g) were stirred in 100 mL of deionized water at 60 °C, 120 rpm until the solution became homogeneous. The sodium alginate solution waited 24 h at room temperature for the gases trapped in the solution to release. Fe-based particles (1 g) were added into 20 mL of the homogeneous and degassed sodium alginate solution. The Fe particles were homogenized in the sodium alginate solution by using an ultrasonic homogenizer (model: UP200HT Hielscher) for 30 min. From the homogenized solution, 100 microliter droplets were added in 50 mL of 0.1 M CaCl_2_ solution in a magnetic stirrer. After adding 20 mL of sodium alginate/Fe solution completely, it was stirred for 1 h in CaCl_2_ solution at room temperature. After 1 h, the gels were filtered and waited 1 day for gelatin to occur completely. The obtained gels were added to 50 mL, 2% acetic acid solution containing 2 g of chitosan and stirred for 1 h. The gels coated with chitosan were filtered and washed 7 times with deionized water to neutralize the pH. Produced chitosan-coated Fe-based particles doped hydrogels were used for recovery of Nd from wastewater.

### 2.4 Adsorption Experiments

The solution contains 100 mg L^–1^ Nd^3+^ ion was prepared by dissolving a sufficient amount of Nd_2_(SO_4_)_3_ in deionized water. All adsorption experiments were performed with 50 mL solution, overhead stirrer (model: OS20-Pro, Dlab), and 500 rpm stirrer speed. The experimental parameters, which are given in Table 1, were used to determine the optimum adsorption conditions. The temperature of the experimental setup was adjusted using a thermostat (model: GPWB-240 V, PolyScience), and pH values were adjusted using 0.1 M NaOH and 0.1 M HCl. In Experiment 1, the time was chosen as 300 min, and samples were taken from the solution at regular intervals. As will be explained in the following sections, the adsorption kinetics were tried to be understood and it was seen that the system reached equilibrium at the 180th minute. In the other 6 experiments, the time was chosen as 180 min. The adsorption efficiency was calculated using eq 2:^21^
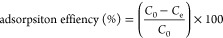
2where *C*_0_ is the initial Nd^3+^ ion concentration (mg L^–1^), and *C*_e_ is the Nd^3+^ ion concentration (mg L^–1^) in the solution at the equilibrium of the system. The adsorption capacity of the adsorbent was calculated using eq 3:^21^
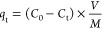
3where *q*_t_ is the adsorption capacity at a certain time (mg g^–1^), *C*_0_ is the initial concentration (mg L^–1^), *C*_t_ is the concentration of the solution at a certain time (mg L^–1^), *V* is the volume of solution (L), and *M* is the amount of adsorbent (g).

**Table 1:** Experiments To Determine the Optimum Adsorption Conditions

	experiment codes						
parameters	1	2	3	4	5	6	7
amount of adsorbent (g)	0.2	0.2	0.2	0.2	0.2	1	1
pH	5.55	3.55	7.55	5.55	5.55	5.55	5.55
temperature (°C)	25	25	25	55	75	25	75

### 2.5 Adsorption Kinetics and Isotherms

The kinetics of the adsorption study was deduced by taking samples from the solution at different time intervals from experiment 1 that actualized with 0.2 g of the adsorbent amount, 5.5 pH, and 25 °C temperature. The most commonly used theorems to explain adsorption kinetics are pseudo-first-order equations, pseudo-second-order equations, intra-particle diffusion models, and the Elovich equation.^28^ Adsorption kinetics can be expressed by these 4 different approaches. The compatibility of 4 different models with the experimental data obtained was examined separately. With eq 3, adsorption kinetics can be explained with the linearized version of the pseudo-first-order eq 4:^29^
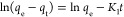
4

In this equation, *q*_e_ and *q*_t_ represent the adsorption capacity at equilibrium and “*t*,” respectively, (mg L^–1^), *K*_1_ is the equation constant, and *t* is the time (min). The pseudo-first-order equation gives the rate of change of ion adsorption with time. *K*_1_ and *q*_e_ values were calculated by plotting ln(*q*_e_ – *q*_t_) versus time. The linearized version of the pseudo-seconder-order (eq 5) is fitted to the experimental adsorption data:^29^
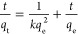
5

The variables here are the same as the pseudo-first-order equation. In adsorption studies that comply with the pseudo-seconder-order equation, it is accepted that the rate limiter is chemisorption. *K*_2_ and *q*_e_ values were calculated by plotting the change of *t*/*q*_t_ value to time. The intraparticle diffusion model was calculated with eq 6:^29^
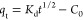
6

The *K*_d_ value was calculated by plotting the variation of *q*_t_ with *t*^1/2^. The Elovich equation was calculated using eq 7:^29^
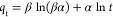
7Here, β (mmol/g) is the deadsorption constant, and α (mmol/t g) is the adsorption constant. The variations of Elovich equations are calculated by plotting *q*_t_ versus ln *t*. The Elovich model is used when describing heterogeneous and chemisorption processes.

Adsorption isotherms are important to explain the interaction between the adsorbent and adsorbance. The relationship between isotherm models and experimental data was examined. In this study, it was seen that the ″Langmuir″ and ″Freundlich″ isotherm models were in better agreement with the experimental data. Eq 8 examined the agreement between experimental data and Langmuir isotherm:^30^
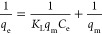
8
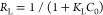
9Here, *C*_e_ is the equilibrium concentration (mg L^–1^), *q*_m_ is the maximum adsorbent capacity of the adsorbent (mg g^–1^), and *K*_L_ is the Langmuir constant. For adsorption studies in accordance with the Langmuir model, it can be thought that homogeneous adsorption occurs on the adsorbent surface and in the monolayer. Also, eq 9 represents the Langmuir separation factor (*R*_L_). The *R*_L_ number reflects the nature of adsorption as either unfavorable (if *R*_L_ > 1), linear (if *R*_L_ = 1), favorable (if 0 < *R*_L_ < 1), or irreversible (if *R*_L_ = 0).^31^ Furthermore, the relationship between the Freundlich isotherm and experimental data can be examined with eq 10:^30^
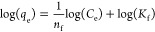
10Here, unlike the Langmuir isotherm, *K*_f_ is the Freundlich constant, and *n*_f_ is the Freundlich isotherm constant, which gives an idea about the adsorption feasibility. Also, typical adsorption is suggested if the value of 1/*n*_f_ is less than 1, but if *n*_f_ equals to 1, the partition between the two phases is concentration-independent.^31^ To explain both isotherms with experimental data, different experiments were accomplished with 1 g of adsorbent, 5.5 pH, 75 °C, and 180 min. These parameters were kept constant and the initial Nd^3+^ concentration was changed to 133, 100, 66, 33, and 16.5 ppm. The results obtained were inserted into the equations in both models and the 1/*q*_e_ versus 1/*C*_e_ change was plotted for Langmuir, and the ln *q*_e_ versus ln *C*_e_ change was plotted for the Freundlich model. Furthermore, *K*_L_ and *K*_f_ were calculated.

### 2.6 Thermodynamic Considerations

Gibbs free energy change (Δ*G*^0^) of adsorption studies was calculated by eqs 11 and 12; also, Δ*H*^0^ and Δ*S*^0^ were calculated by eq 13:^22^
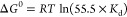
11
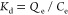
12
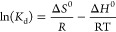
13

The value of *K*_d_ in the equation represents the equilibrium constant of adsorption. By determining Δ*G*^0^, it is possible to have an idea about whether the system occurs spontaneously or nonspontaneously. Δ*H*^0^ and Δ*S*^0^ were calculated via plotting *K*_d_ vs 1/*T*.

### 2.7 Deadsorption Studies

Recovery of adsorbed metal ions is important economically. Therefore, a certain amount of adsorbent was mixed with 0.1 M H_2_SO_4_ 0.1 M HCl, 0.1 M HNO_3_, 0.1 M tartaric acid, 0.1 M glycolic acid, and 0.1 M ascorbic acid, separately, for 3 h at room temperature with the magnetic stirrer. Deadsorption efficiencies for different media were calculated by eq 14;
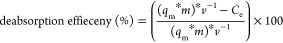
14where *q*_m_ represents the adsorption capacity of the adsorbent (mg g^–1^), *m* represents the amount of adsorbent (g), and *v* represents the solution volume (L).

### 2.8 Characterization

The chemical structure of the Fe-based particles produced was determined by XRD (Rigaku Miniflex, Cu *K*α, 10° ≤ 2Θ ≤ 90) phase analysis. SEM/EDS (model: FEI Quattro Analytical Scanning Electron Microscope, Thermo Fisher) was used for the morphology and surface elemental analysis of Fe-based particles, and FESEM (model: JEOL JSF-7000F Field Emission SEM) analysis was used to understand the morphology and oxide thickness of the particles. SEM and FESEM samples were prepared by dropping the particles suspended in alcohol onto an Al-based substrate and drying them in a vacuum atmosphere without applying heat. DSC (N_2_ atmosphere, 10 K/min) (204f1, Netzch) analysis was performed to understand the thermal properties of the synthesized Fe-based particles, and SEM/EDS analysis was performed for the morphology and surface elemental analysis of the sample obtained after the DSC analysis. The viscosity of sodium alginate and Fe-based particles added to sodium alginate solution was determined by a U-tube viscometer (Paragon Scientific LTD) and eq 15:^32^
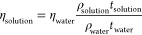
15

By comparing the time needed for a liquid to flow between two lines drawn on a viscometer under the influence of gravity to the time needed for a reference fluid with known viscosity and density to flow between the same two lines at the same temperature, the viscosity was determined.^32^ As a reference solution, water was employed. The gravity bottle was used to gauge the solution densities. Every measurement was made at 20 °C. At 20 °C, water viscosity was supposed to be 1.002 mPa s.^33^ The water adsorbed by the gels during production was calculated with eq 16:
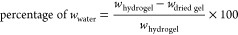
16

After the gels were dried, they were kept in deionized water and the weight gain was calculated with eq 17:
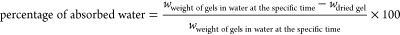
17

After drying the gels produced at 80 °C for 2 h, their chemical structure was determined by FTIR (Bruker) analysis and their thermal properties were determined by DSC (N_2_ atmosphere, 10 K/min) (204f1, Netzch) analysis. The magnetic properties of the produced chitosan-coated Fe-doped gels were determined by VSM (at 25 °C). Nd^3+^ ion concentration in the solution was determined by inductively coupled plasma mass spectrometry (ICP-MS).

## 3 Results and Discussion

### 3.1 Fe-Based Particle Characterization

#### 3.1.1 XRD Analysis of Fe Particles

**Figure 1:**
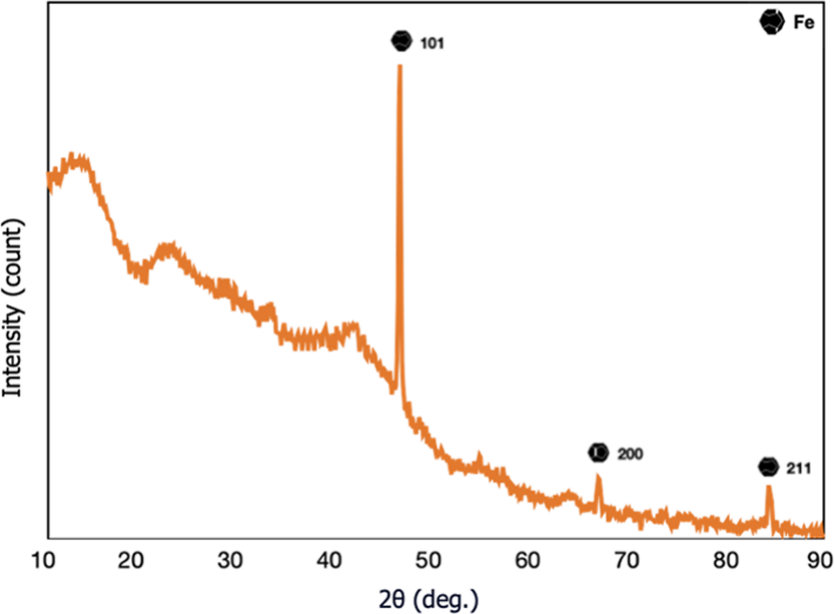
XRD pattern of synthesized Fe particles.

XRD phase analysis of Fe-based particles is given in Figure 1. Characteristic Fe peaks were observed at 2θ = 46.0284, 66.8581, and 84.3150° (ICDD number: 96-900-6601). In other reduction studies of Fe on polyols, it has been shown that the oxide layer is formed but cannot be determined by XRD, also the oxide layer reaches the limit that can be determined by XRD with the increasing surface area when the particle size is reduced below 100 nm.^19,20^ In this study, oxide phases could not be observed, due to the particle size. The presence of the oxide layer was determined by SEM/EDS analysis in the following sections. The crystallite size of the synthesized Fe particles was calculated by modified Debye–Scherrer (MDS) and Williamson–Hall analysis (W–H) based on the uniform deformation model (UDM). They are given in Supporting Information Figure S1. The crystallite size was calculated as 34 nm by W–H analysis based on a uniform deformation model. MDS analysis was performed to compare the W–H analysis. Crystallite sizes of the synthesized Fe particles were approximately 21 nm. As the lattice strain was not taken into account, as expected, the crystallite size values determined by the MDS approach differ from those determined by the W–H method. The MDS approach calculates smaller crystallite sizes than the W–H method because the lattice contains tensile stress. Similar findings were reported elsewhere.^34−38^ Using crystallite sizes derived from W–H analysis and the Williamson–Smallman (W–S) analysis, the average dislocation density was calculated (see Supporting Information). 8.65 × 10^–4^ δ was determined to be the average dislocation density of the produced Fe-based particles. Due to a combination of nanoparticles and uncompensated spins along the dislocation lines, it is known that there is a relationship between magnetic properties, crystal size, and dislocation density.^39^ Also, small dislocation density values show a high degree of crystallization.^40^ The dislocation density was calculated in order to better understand the magnetic properties of the gels we produced and to show that the crystallization takes place at high values.

#### 3.1.2 SEM/EDS Analysis of Fe Particles

First, SEM/EDS analysis was performed immediately after the particles were reduced and before the gradual cleaning process to understand the mechanism of reduction. SEM/EDS analysis of unwashed particles is presented in Supporting Information Figure S2. The presence of salt phases (light color) in SEM images and EDS results also show the presence of Na-based residue phases. The distribution of elements and expected phases in the unwashed particles are given in Table 2. It has been observed that Na_2_CO_3_ may indeed be formed in the system, which is compatible with the literature claim that ethylene glycol gets oxidized to CO_3_^–^ and the surfaces of particles coated with Fe_3_O_4_ due to the EDS result. The expected phases are fully coupled, with 4.5% C atoms remaining; this is thought to be due to impurity.

**Table 2:** EDS Results of Unwashed Particles and Expected Phases

EDS results					
element	percentage (%)	expected Phases	Na_2_(SO)_4_	Na_2_CO_3_	Fe_3_O_4_
C	9	Na_2_(SO)_4_		4.5	
O	52.5	Na_2_CO_3_	26.4	13.5	12.3
Na	22.2	Fe_3_O_4_	13.2	9	
S	6.6		6.6		
Fe	9.1				9.1

**Figure 2:**
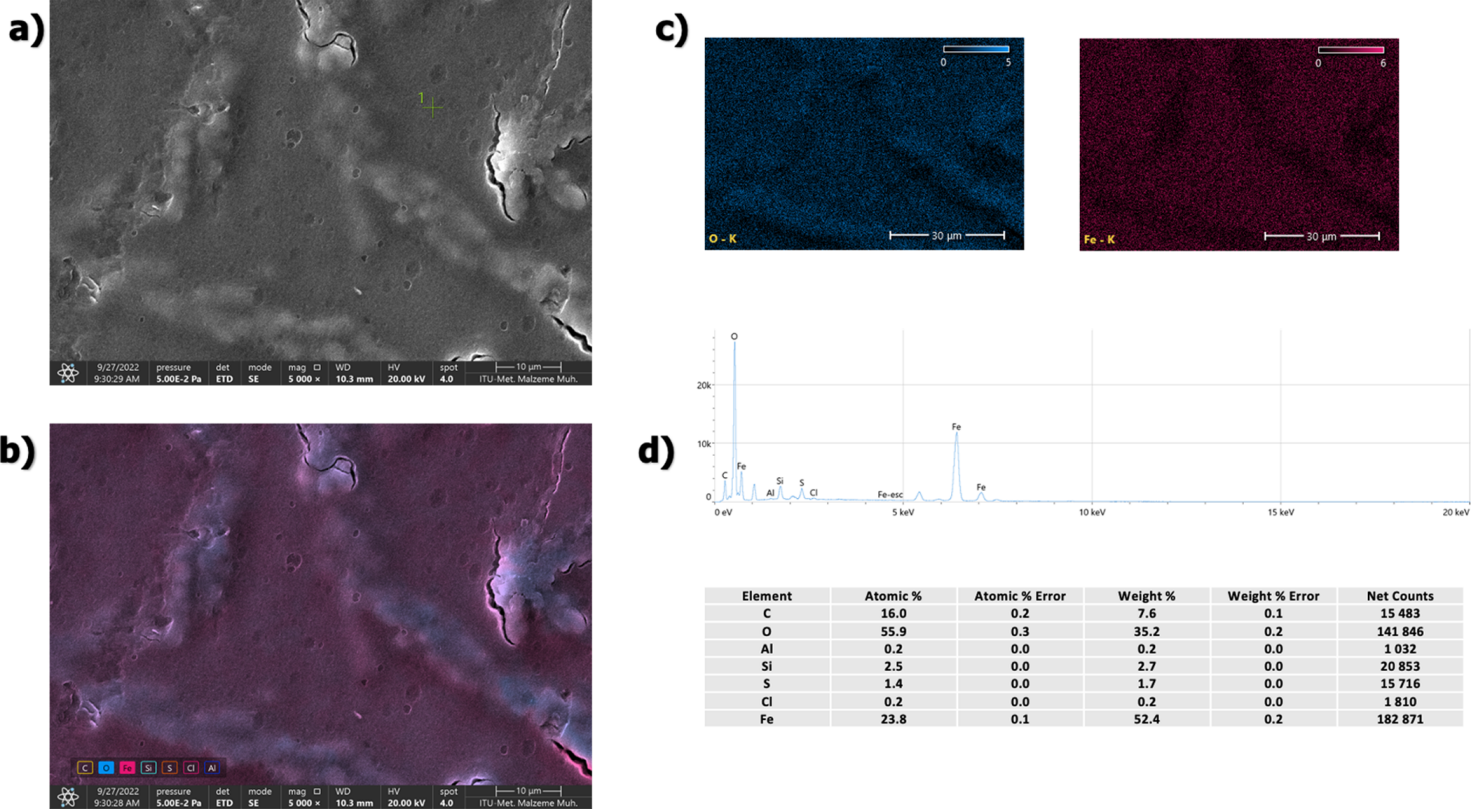
SEM/EDS analysis of washed particles; (a) ×5000 magnification, (b) ×5000 magnification/quant map of Fe, (c) EDS maps, (d) EDS data and elemental compositions.

**Figure 3:**
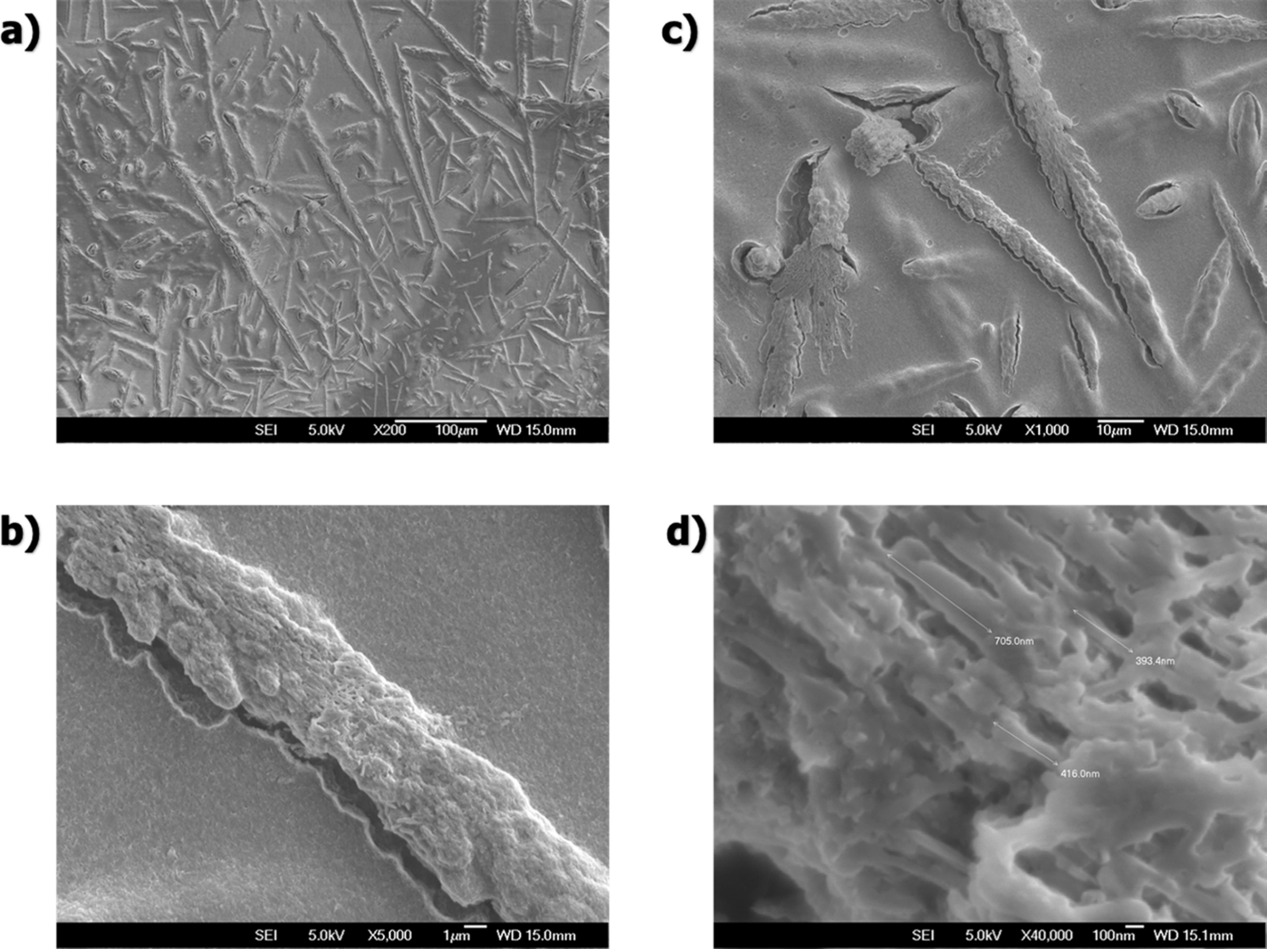
FESEM analysis of particles; (a) ×200, (b) ×1000, (c) ×5000, (d) ×40,000 magnification.

SEM/EDS analysis of washed particles is given in Figure 2. As can be seen in the SEM images, the particles agglomerated together during drying. It is seen that there is growth in a certain direction with the magnetic field effect you have during the drying phase. As a result of the EDS analysis, no other significant element except oxygen and iron was observed in the structure (the carbon in the EDS results is fused from the coating while preparing the SEM sample and impurities). In regions where the presence of oxygen and therefore the oxide layer is dense, the agglomeration is more significant. The results of the EDS quant map and EDS map confirm (see Figure 2c,d) that the dense regions of the oxide layer come together and grow in certain directions. FESEM analysis was used to determine the thickness of the oxide layer and the size of the particles (see Figure 3). FESEM images show that the regions where the oxide layer is dense are agglomerated in certain directions during the drying phase. It was observed that the particles were in the form of rods with an average length of 400 nm, and the thickness of the oxide layer was 16 nm on average. In other studies, using FeCl_2_ precursor solution, it has been reported that the reduced Fe particles are in a cube shape.^19,20^ It has been reported that applying a magnetic field to Fe nanoparticles synthesized in the cubic form during reduction causes the particles to grow in the form of chains at a certain level.^19^ A magnetic stirrer and heater were used in this study. The magnetic field created by the magnetic stirrer was thought to cause the particles to grow in rods rather than cubes.

#### 3.1.3 DSC Analysis of Fe Particles

**Figure 4:**
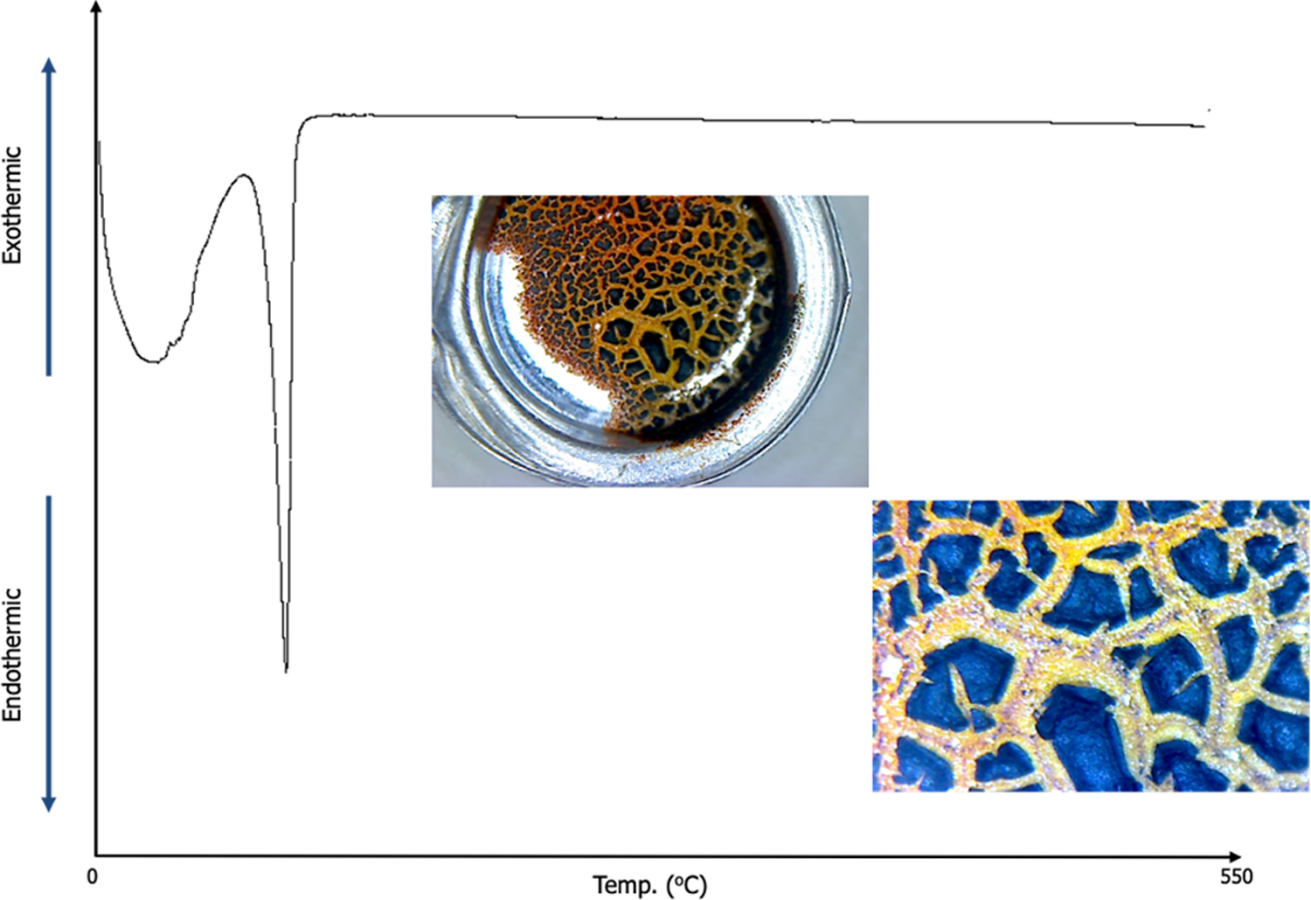
DSC diagram of Fe particles and optical image of particles after DSC analysis.

**Figure 5:**
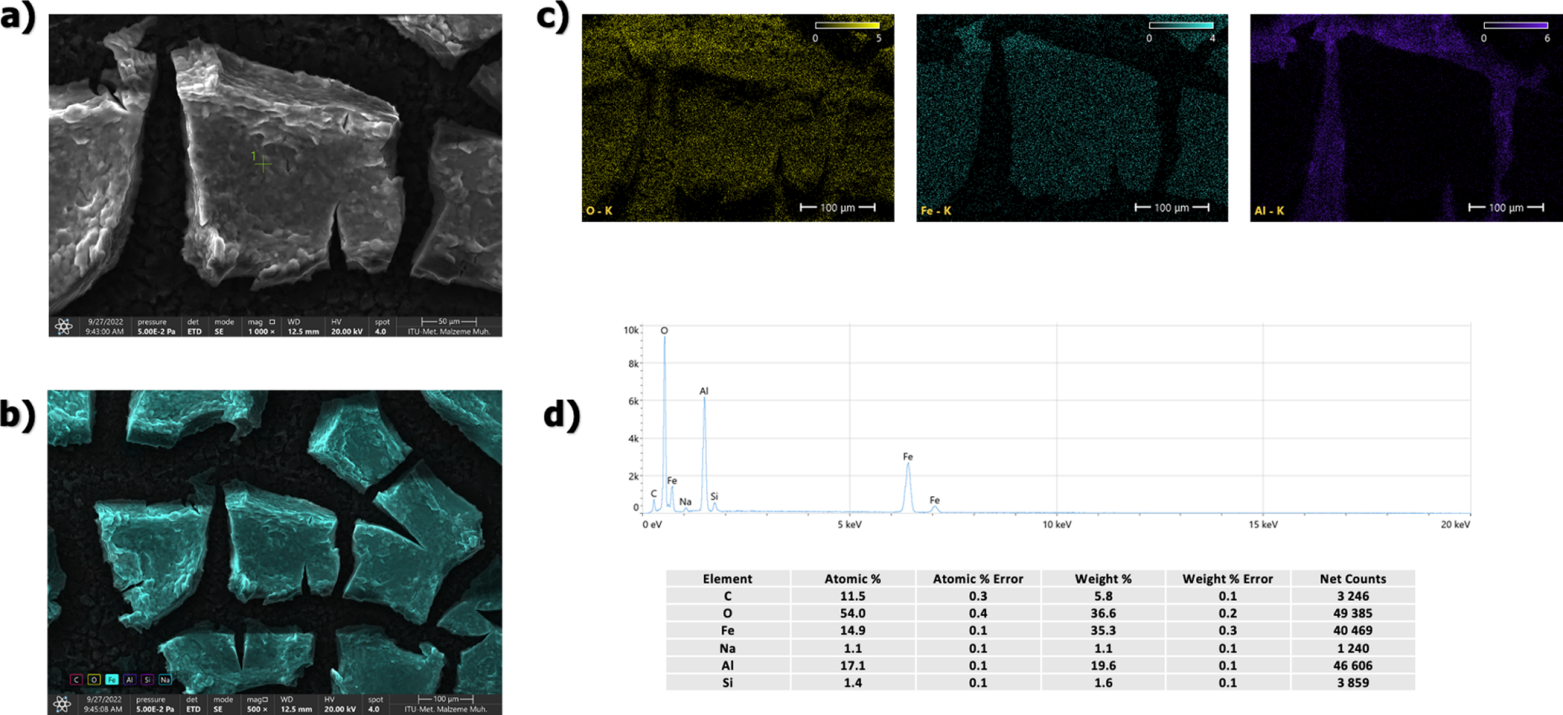
SEM/EDS analysis of Fe particles after DSC analysis; (a) ×1000 magnification, (b) ×500 magnification/quant map of Fe, (c) EDS maps, (d) EDS data and elemental compositions.

DSC analysis was performed to understand the thermal behavior of Fe particles (see Figure 4), and then, SEM/EDS analysis of the obtained structure was performed (see Figure 5). The first, broad peak indicates the dehydration of the water remaining on the particles during the washing phase, while the second sharp peak indicates the gelatinization endotherm of Na-based salts.^41^ There is not observed crystallization peak of Fe, so the reduction temperature was enough to synthesize crystalized Fe particles. Figure 5 also shows the SEM/EDS analysis of the particles obtained after DSC analysis. It has been observed that the particles agglomerated with the effect of temperature and formed macro-sized particles. EDS maps also show that the particles are based on Fe. Al peaks come from the substrate. Oxygen was evenly distributed; however, it was observed that metallic Fe was present in some regions and did not form an oxide phase. It was thought that this effect was related to diffusion and that it was effective with the diffusion behavior of iron oxide under high temperatures.

### 3.2 Formation and Characterization of Hydrogels

**Figure 6:**
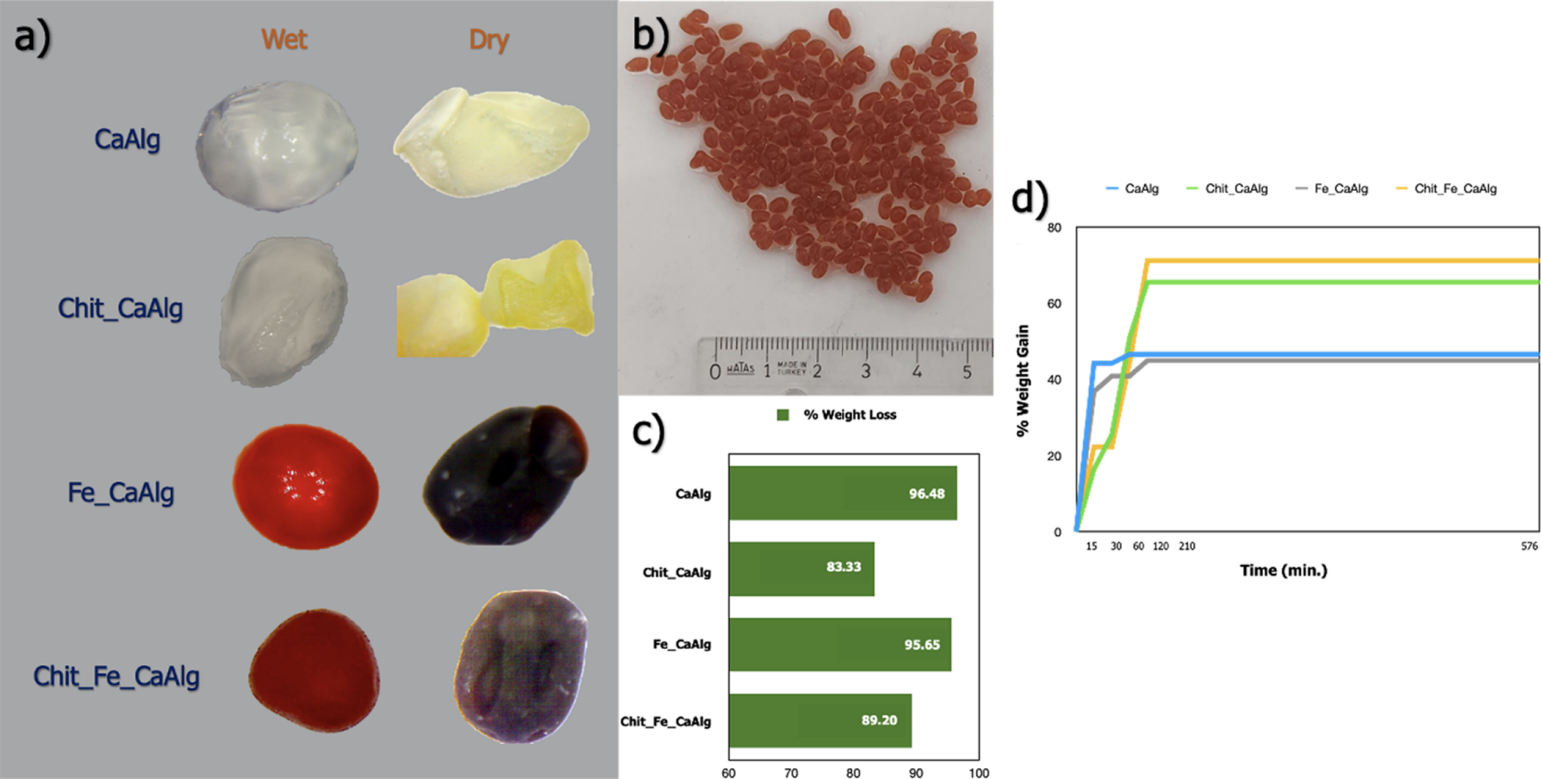
Wet and dry gels (a), synthesized Fe particle doped hydrogels (b), weight losses after 3 h, at 80 °C (c), and weight gain at deionized water (d).

The wet and dry states of the produced hydrogels are presented in Figure 6a and Fe-based particle-doped chitosan coated hydrogels are given in Figure 6b. It has been observed that the coating of alginate gels with chitosan affects the deterioration of the spherical morphology. The average diameter of Fe-based particle-doped hydrogels is 4.04 mm. Furthermore, it has also been observed that chitosan-coated and uncoated gels were dried at 80 °C for 3 h in a standard atmosphere, disrupting their spherical morphology. In addition, coating the gels with chitosan caused a change in the +b* direction on the color coordinate.

The pH of the sodium alginate (NaAlg) solution and the NaAlg solution doped with Fe nanoparticles was measured as 4.29 ± 0.02 (at 21.6 °C). The pH of the solution containing (2% v/v) acetic acid and containing chitosan was measured as 3.76 (at 21.1 °C). The viscosities of SA solution and SA solutions containing Fe-based particles were measured as 173.06 and 64.36 mPa S, respectively. The reason for this is thought to be the effect of the zeta potential of Fe-based particles on the solution. The particles we produce are thought to have an outer surface in the form of Fe_3_O_4_. Fe_3_O_4_ particles have a negative value in the pH 3–5 band,^42^ and this negative value interacts with the negatively charged −COO groups and repels each other. Furthermore, the change in weight of all gels after the drying step was calculated (see Figure 6c). This change in their weight gives an idea of how much water they absorb during the production phase. It has been observed that coating alginate hydrogels with chitosan reduce the amount of water absorbed. Coating alginate beads with chitosan at pH 3.76 causes protonation of carboxyl groups at low pH and some deterioration of the rigid structure.^43^ For this reason, gels coated with chitosan absorb less water during the production step. Furthermore, some deterioration of the spherical structure is also due to this reason. To examine the water absorption behavior of the gels, they were kept in deionized water for 576 h and the weight change was measured at different intervals (see Figure 6d). While the rate of weight gain was relatively high in the first 15 min, this rate decreased in the following time. It is predicted that this effect is related to the diffusion rules, and as the water concentration in the gels increases, the diffusion rate decreases over time. It was observed that the gels reached equilibrium within 120 min and maintained the change in weight at the end of the 576th minute. The most important point in the water absorption behavior is while the chitosan-coated gels absorb less water during the production step, chitosan-coated gels absorb more water in the aqueous environment than the nonchitosan-coated gels due to the increase in the number of −OH groups increasing with chitosan and the increase in secondary bond interactions between water and −OH groups. The addition of Fe-based particles to the structure did not have a striking effect on both water release and water absorption behavior. As explained in the thermal analysis of the gels section, Fe particles were effective on the amount of nonfreezing water content.

#### 3.2.1 FTIR Analysis of Hydrogels

**Figure 7:**
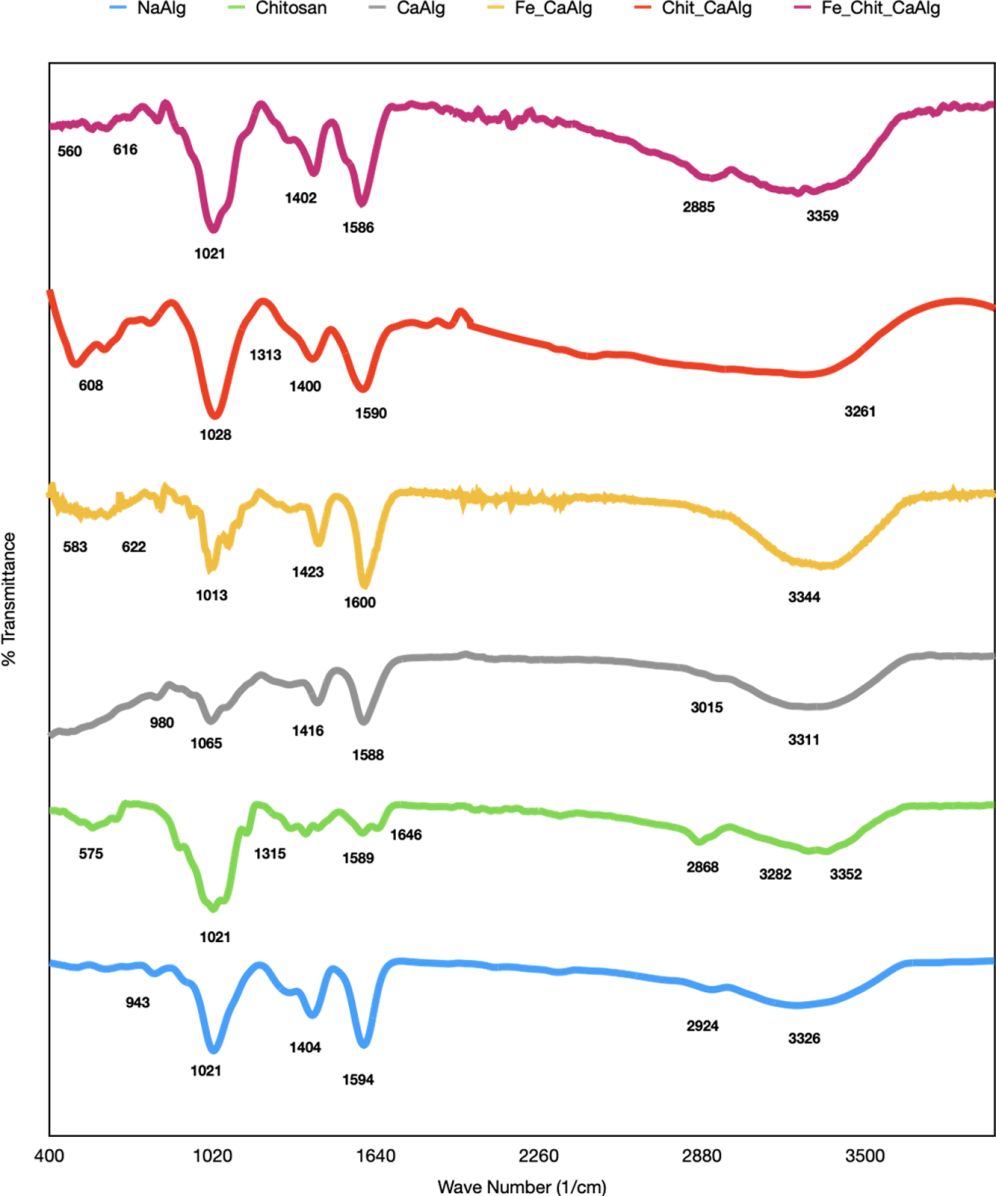
FTIR results of hydrogels.

To determine the structure of the bonds in the synthesized gels, FTIR analysis was performed, and the results are presented in Figure 7. NaAlg showed absorption peaks, referring to the hydroxyl, ether, and carboxyl groups. The absorption peak in the range of 3000–3500, and a peak of 3326.101 cm^–1^ refers to the O–H bonds in the chemical structure of NaAlg.^44^ Aliphatic C–H vibrations were observed at 2924.818 cm^–1^; furthermore, asymmetric and symmetric stretching vibrations of the carboxylate salt ion were observed at 1594.791 and 1404.492 cm^-1^,^45^ respectively. The peak observed at 1021.825 cm^–1^ refers to the C–O vibration of the pyranose ring, and the peak observed at 943.223 cm^–1^ refers to the C–O stretching formed by the C–C–H and C–O–H deformation.^46^ It is observed that the absorption peaks caused by the chemical structure of calcium alginate (CaAlg) shift relative to NaAlg. It is seen that aliphatic C–H vibrations shifted to 3015.83 cm^–1^ and asymmetric–symmetrical carboxylate ion vibrations to 1588.586 and 1416.902 cm^–1^. Furthermore, it is determined that the C–O vibration of the pyranose ring’s peak shifted to 1065.262 cm^–1^, and the C–O stretching, formed by the C–C–H and C–O–H deformation, occurred at 980 cm^–1^. Chemical property differences between the Ca^2+^ ion and the Na^+^ ion, such as the atomic radius, have caused such absorption peak shifts. Moreover, these peaks in CaAlg were narrower than the peaks in NaAlg’s IR spectrum. This narrowing means that the “Egg-Box” structures formed, limiting molecules’ mobility under the IR spectrum.^47^ Narrowing was observed in the 3000–3500 cm^–1^ band of CaAlg. It is known that divalent metal ions, such as Ca^2+^, have a chelating effect on the alginate structure. The narrower band in calcium alginate is due to the resulting reduction in hydrogen bonding between hydroxyl functional groups as a result of the chelating effect.^46^ The addition of Fe-based particles into the CaAlg hydrogels caused the carboxylate bonds to shift to the right (1600.996 and 1423.108 cm^–1^), while the C–O stretching and C–O stretching in the pyranose ring, formed by the C–C–H and C–O–H deformation bonds, shifted to the left (1013.551 and 941.151 cm^–1^). It is thought that the reason is the magnetic field of Fe-based particles has also affected Ca^2+^ ions and (CO_2_)^−^ groups. Furthermore, the peaks between 580 and 600 cm^–1^ wavelengths are thought to be related to the Fe–O bond.^12^

In the IR spectrum of chitosan, absorption peaks were observed at 3352.991 and 3282.663 cm^–1^ wavelengths. These peaks refer to the stretching of O–H and N–H bonds.^48^ The absorption in the 2868.969 cm^–1^ wave number is referred to as the aliphatic C–H stretch, which is characteristic of typical polysaccharides.^49^ The bands at roughly 1646.503 cm^–1^ (C=O stretching of amide I) and 1315.547 cm^–1^ (C–N stretching of amide III) provided evidence of the existence of residual *N*-acetyl groups.^48,50^ The absorption peak (1588.586 cm^–1^) has occurred via N–H bending of primary amine, and 1421,039 and 1375 cm^–1^ absorption peaks refer to the CH_2_ bending and CH_3_ symmetrical deformations, respectively.^48−50^ C–O–C bonds’ give a peak at 1148.001 cm^–1^, while C–O bonds’ stretching peaks have occurred at 1061.125 and 1021.825 cm^–1^.^48^ chitosan’s absorption peak at 1646.503 cm^–1^, which is related to the amine group, shifted to the left in Chit_CaAlg and Fe_Chit_CaAlg’s IR spectrum. Chitosan’s N–H absorption peak at 1588,586 cm^–1^ disappeared at both Chit_CaAlg and Fe_Chit_CaAlg’s IR spectra. These mean that there are interactions between alginate’s (CO_2_)^−^ and chitosan’s (NH_3_)^+^ groups.^51^ The absorption peak range between 3000 and 3500 cm^–1^ gets broad, when alginate structure coated chitosan, because of increasing O–H and N–H bonds.

#### 3.2.2 DSC Analysis of Fe Particles and Hydrogels

**Figure 8:**
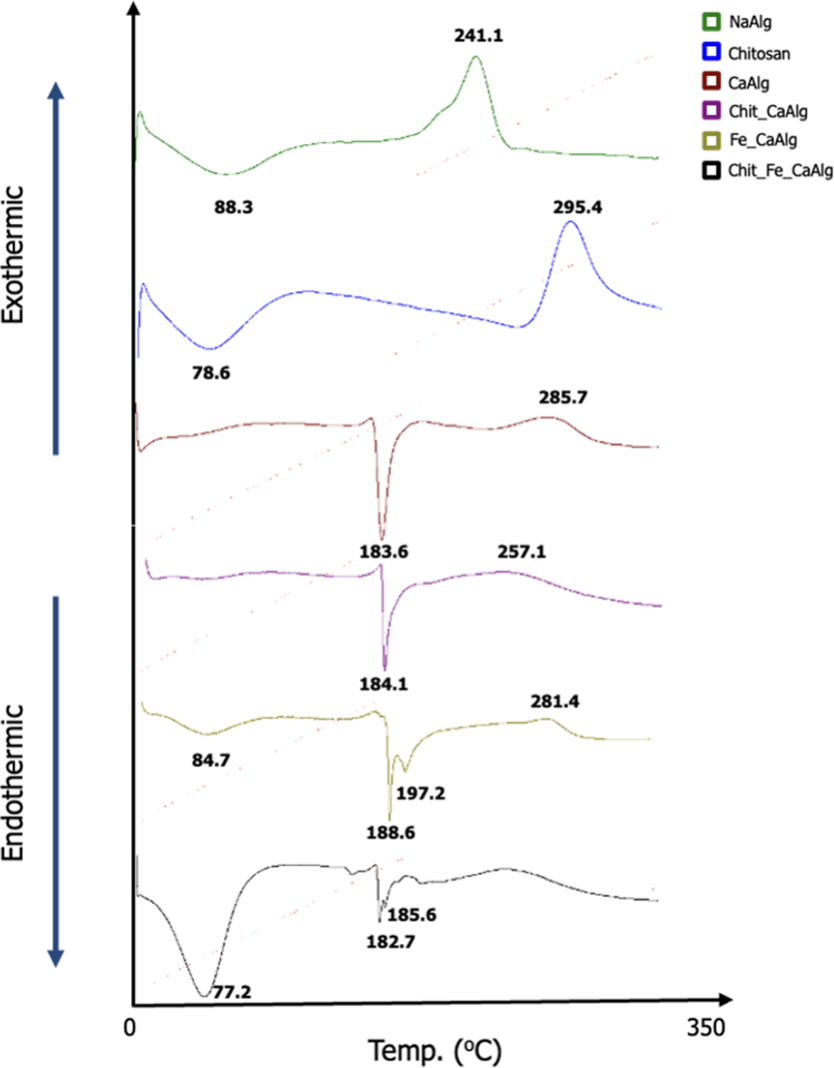
DSC diagrams of gels.

DSC analysis was performed to examine the thermal behavior of the synthesized gels and to observe the changes in their chemical structures, and the results are presented in Figure 8. Sodium alginate gives the first peak at 88.3 °C, which attributes to the dehydration of the structure; furthermore, the peak at 241.1 °C refers to the thermal degradation of NaAlg.^52^ Also, while the chitosan dehydration peak occurs at 78.6 °C, the decomposition peak was at 295.4 °C.^53^ Different metal alginates thermodynamically contain three different types of water; free water, weakly bound water, and nonfreezing-strong bound water.^52^ DSC analysis was performed after all hydrogels and powders were dehumidified. While it is easy to separate free and weakly bound water from the structure of gels and powder, it is difficult to remove strongly bound-nonfreezing water from the structure. The strong nonfreezing water in the gels is a function of the metal ion and the amount of nonfreezing water increases as the ionic diameter increases.^52^ Since the diameter of the Na^+^ ion is larger than the Ca^2+^ ion, the amount of water that is strongly bonded in its structure is greater. For this reason, while the dehydration peak occurs in the DSC diagram of NaAlg, the dehydration peak does not occur in the structure of CaAlg and Chit_Ca_Alg. When Fe particles were doped to the gels, dehydration peaks were observed to occur again. The reason for this was interpreted as the interaction of Fe particles with (CO_2_)^−^ groups, as seen in the FTIR analysis, and the increase in the amount of water trapped in the structure by opening the distance between the chains with doping. In addition, while the dehydration peak of Fe_Ca_Alg was close to that of NaAlg, Chit_Fe_Ca_Alg was close to the dehydration peak of chitosan. As shown in Figure 6, chitosan-coated gels were thought to absorb more water and show closer thermal behavior to chitosan.

Cross-linking alginate with calcium increases the decomposition temperature. It is known that cross-linking with Ca^2+^ ions increases alginate’s resistance to thermal oxidation.^54^ Fe particle doping into the CaAlg structure did not have a striking effect on the thermal oxidation behavior. Coating the gels with chitosan decreased the thermal oxidation resistance because the interaction between alginate and chitosan started at relatively low temperatures.

A sharp peak was observed around 180 °C in all calcium alginate gels. The sharp endothermic peak around 180 °C refers to the disruption of the interaction between Ca^2+^ and (CO_2_)^−^and even indicates the presence of “Egg-Box” structures in gels.^54^ The presence of a second sharp peak was observed in the Fe-doped gels, and the interactions between Fe particles and (CO_2_)^−^ groups were seen by FTIR analysis. It has been observed that Fe particles interact with these groups via their magnetic field and strengthen the existence of “Egg-Box” structures.

#### 3.2.3 VSM Analysis

**Figure 9:**
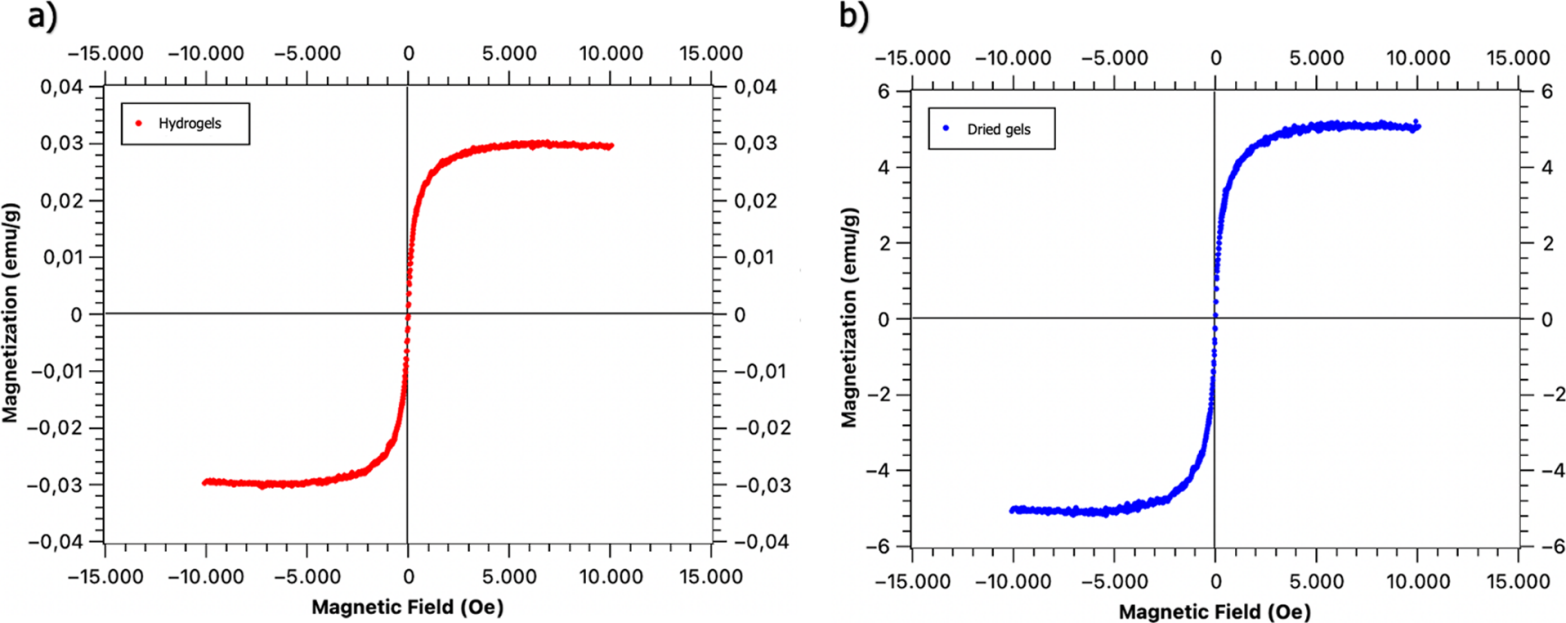
VSM analysis of Fe-doped (a) hydrogel and (b) dried gels.

VSM analysis results of hydrogels and dried gels are given in Figure 9. Hydrogels and dried gels showed ferromagnetic properties and their magnetic saturations were found to be 0.297 and 5.136 emu/g at room temperature, respectively. The difference is due to the very high water content of the hydrogels.

### 3.3 Nd^3+^ Ion Removal from Wastewater

#### 3.3.1 Adsorption Time

**Figure 10:**
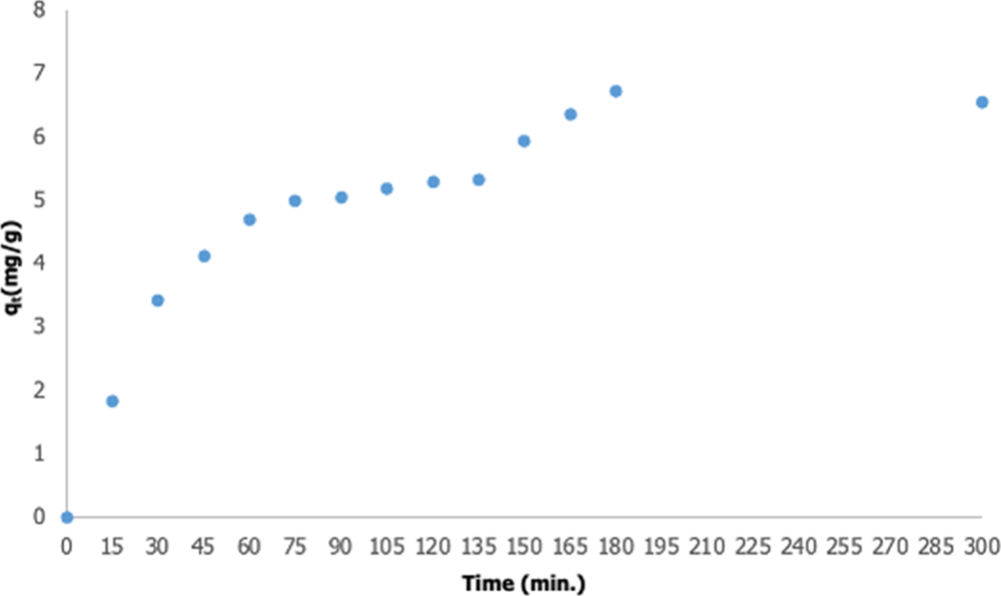
Time-dependent variation of the adsorption capacity of the adsorbent (50 mL solution, 0.2 g adsorbent, pH 5.5, 25 °C).

In adsorption studies, the contact time of the adsorbent and adsorbance should be optimized as it affects the kinetic stability of adsorption.^21^ To determine the adsorption time, the first experiment was carried out with a solution containing 0.2 g of adsorbent and 50 mL of 100 mg/L Nd^3+^ ions at pH 5.5, temperature 25 °C, and 300 min. The time-dependent variation of the adsorption capacity (*q*_t_) of the adsorbent was calculated and is given in Figure 10. The adsorption system reached equilibrium at the 180th minute. After the first experiment, all experiments were done at 180 min. The adsorption capacity (*q*_e_) of the adsorbent at equilibrium was found to be 6.72 mg/g. In literature studies on the removal of heavy metals from wastewater by adsorption using alginate-based magnetic gels, this value was found to be between 200 and 300 mg/g.^55−58^ In this study, the capacity of the adsorbent has been tried to be kept low so that deadsorption can be done easily in an acid medium. In the study of Hamed et al., the adsorptions of Ce^3+^ and Fe^3+^ ions were studied using magnetic alginate-based gels; while HCl acid yielded 20%, HNO_3_ 16.2%, oxalic acid 0%, and citric acid 24.5% in deadsorption studies, EDTA provided 100% efficiency.^21^ Since Nd is a metal with a high economic value, which is in the list of critical metals, the adsorption capacity of the adsorbent has been tried to be kept low by taking into account the deadsorption studies.

#### 3.3.2 Adsorption Kinetics

**Figure 11:**
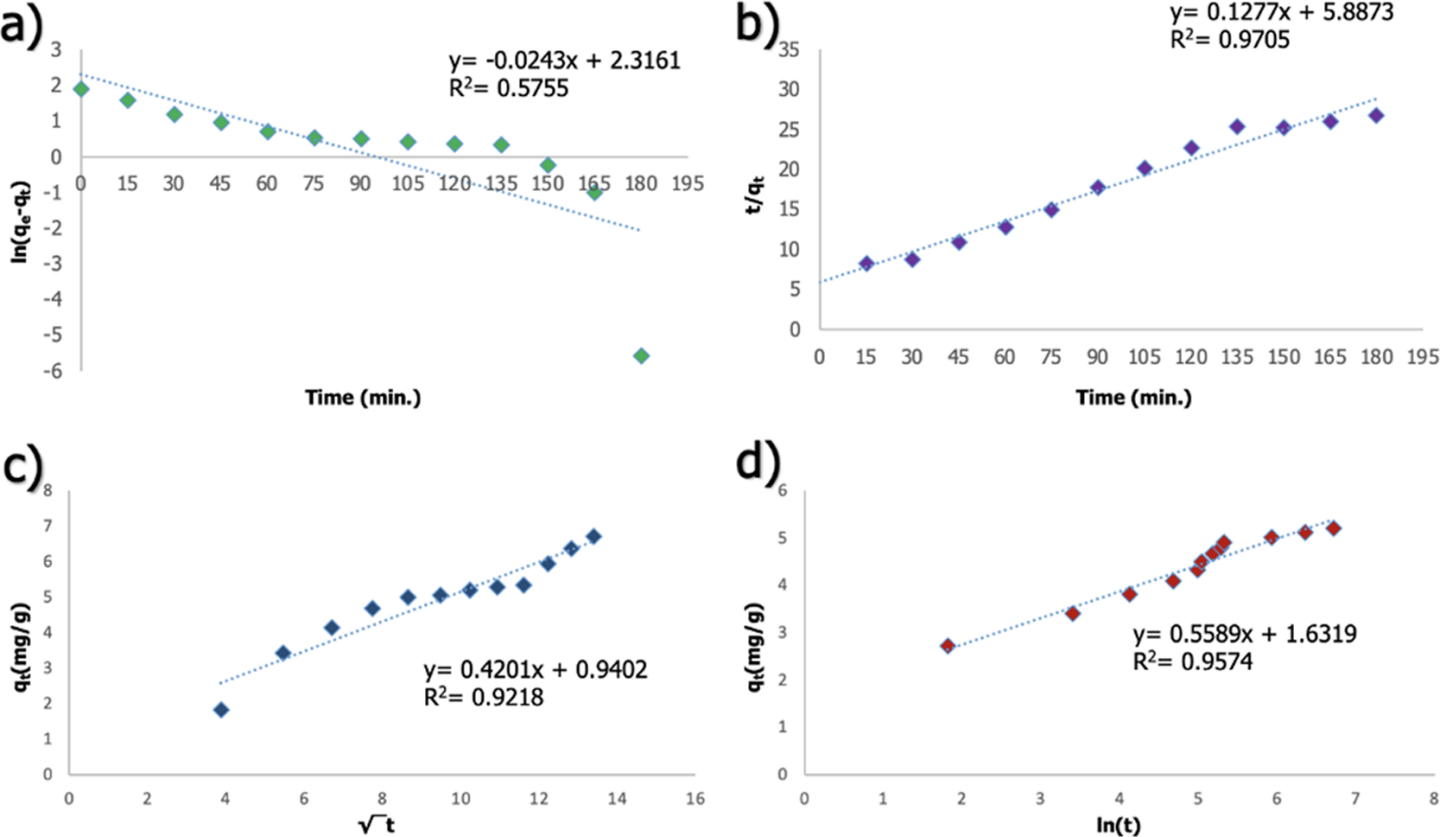
Agreement of the absorption data with the different models; (a) pseudo-first-order, (b) pseudo-second-order, (c) Weber-Moris, and (d) Elovich equations.

**Figure 12:**
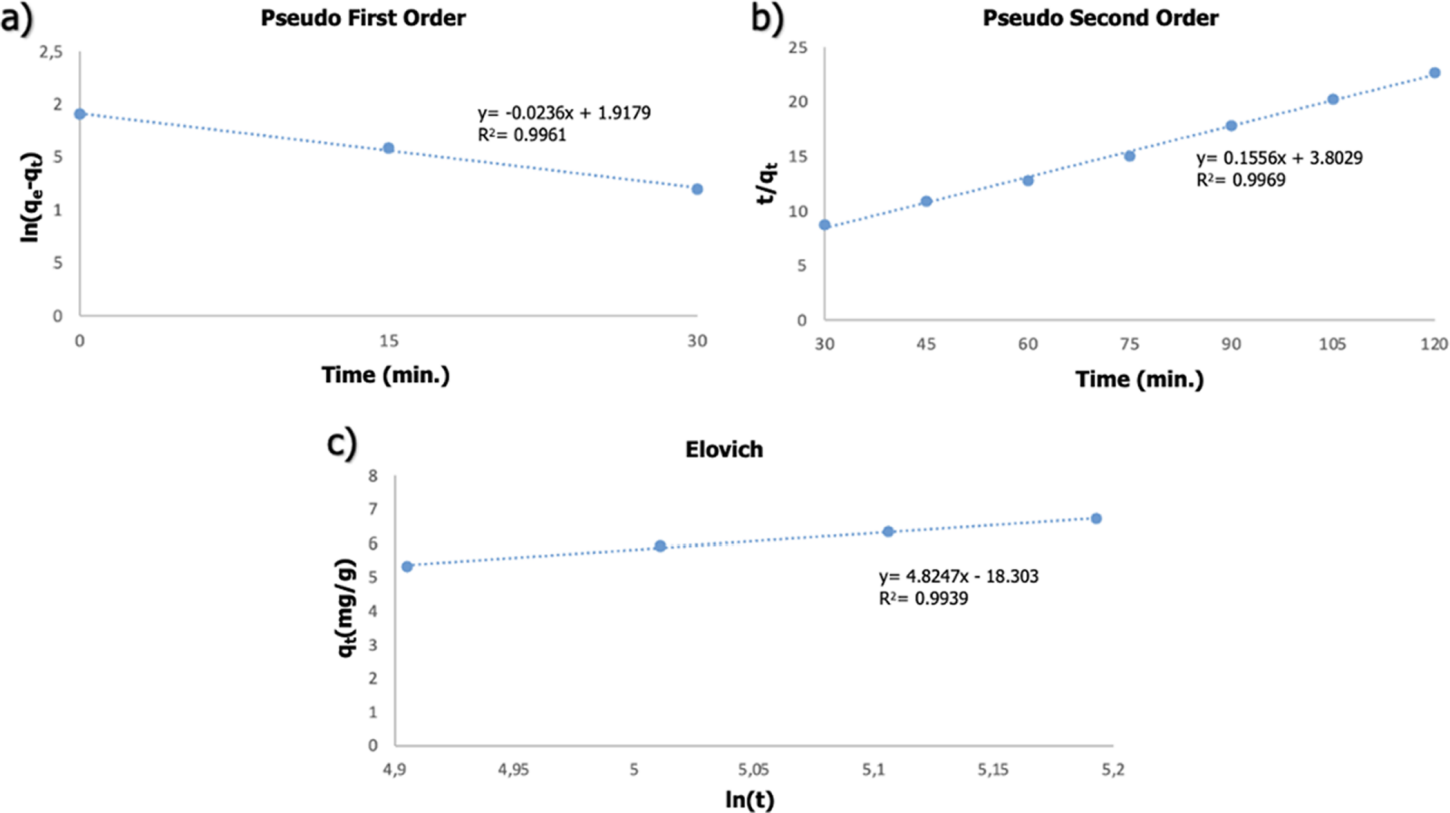
Examination of the kinetics of the system divided into different time intervals; (a) 0–30th, (b) 30–120th, (c) 135–180th minutes.

To determine the optimum reaction parameters in applications with the same operating conditions, the kinetic properties of metal ion adsorption should be determined.^21^Figure 11 represents four different models’ agreement with experimental data. Reaction constants and *R*^2^ values are given in Supporting Information Table S1. *R*^2^ values calculated with four different equations were not greater than the 99% confidence interval. *R*^2^ of the highest value was achieved by the pseudo-second-order equation with 97.05%. Therefore, the general effective mechanism in the adsorption process is chemisorption. However, in adsorption systems, as in this study, it may not be possible for a single mechanism to be effective on the system, and a linear agreement between the experimental data and a kinetic equation may not be possible. In such reactions, more than one limiting factor is effective on the system, the data can be divided into different time intervals, and kinetic analysis can be performed.^59^ The adsorption study was divided into different time intervals, the results are given in Figure 12, and the calculated variables are given in Table 3. The first 30 min of adsorption showed 99.61% compliance with the pseudo-first-order equation. It has been understood that the first minutes of adsorption comply with the pseudo-first-order, due to the high concentration difference between the wastewater and the solution in the hydrogels at the first 30 min of adsorption. Furthermore, the adsorption kinetics exhibited by gels with sufficient saturation between the 30th and 120th minutes comply with the pseudo-second-order. Moreover, weak interactions (magnetic field effect) affect adsorption. The system is chemisorption-controlled in this time interval. After the 120th minute, it was seen that the system complied with the Elovich equation. The Elovich model assumes that the rate of adsorption of solute decreases exponentially as the amount of solute adsorbed increases.^60^ In addition, the Elovich equation is applied to systems with heterogeneous adsorption surfaces of chemical adsorption processes.^61^ While the accumulation of Nd^3+^ on the surface of the adsorbent increases with magnetic separation, the Nd^3+^ concentration in the solution decreases. For these reasons, it was determined that the system followed the Elovich equation after the 120th minute. It was observed that the calculated *q*_e_ values were very close to the experimentally obtained *q*_e_ values (see Table 3).

**Table 3:** Equations Applied and Calculated Variables for Different Time Intervals

0–30th minutes			30–120th minutes			120–180th minutes		
pseudo-first-order			pseudo-second-order			Elovich		
*q*_e_ calculated (mg/g)	*k*_1_ (min^–1^)	*R*^2^	*q*_e_ calculated (mg/g)	*k*_2_ (g/mg min)	*R*^2^	α (mg/(g t))	β (g/mg)	*R*^2^
6.766931854	0.0236	0.9961	6.42673522	0.0063	0.9969	6.7243	0.20726677	0.9939

#### 3.3.3 Effect of pH on Adsorption

Three experiments were carried out with 50 mL of a solution containing 100 mg/L Nd^3+^ ion and 0.2 g of adsorbent, at 25 °C at 3 different pHs (3.5, 5.5, and 7.5) for 180 min. The obtained adsorption efficiencies are given in Table 4. While the adsorption efficiency was calculated as 26.8% at pH 5.5, it was calculated as 17.69 and 17.24% for pH 3.5 and 7.5, respectively. It was thought that increasing H^+^ ions as pH decreased led to decrease in the adsorption efficiency. In literature studies, it was reported that the accumulation of H^+^ ions on the adsorbent surface and the efficiency of the adsorbed metal ions decreased for this reason.^21,56^ In this study, it was observed that the H^+^ ions adsorbed at lower pH values accumulate on the adsorbent surface and decrease the adsorption efficiency. Also, the adsorption efficiency decreased again when the pH of the system was increased to 7.5. The pourbaix diagram was drawn with the “Materials Project” program with the amount of Nd^3+^ and SO_4_^–^ used in the adsorption system^62−64^ and is given in Figure S3. It was observed that Nd did not precipitate as a solid until the pH value was around 10.5. However, along with Nd^3+^ ions, the adsorption of H^+^ ions also takes place. As a result, the pH value increases locally and may cause Nd to precipitate as a solid, or it has been predicted that the produced gels may also result from the positive increase in the zeta potential at high pH. For these reasons, the higher the pH, the lower the adsorption efficiency. It has been deemed appropriate that the pH 5.5 value is the optimum value for adsorption efficiency and that all subsequent experiments should be carried out at pH 5.5.

**Table 4:** Adsorption Efficiency Calculated with Changing pH Values (50 mL, 100 mg/L Nd, 0.2 g of Adsorbent, 25 °C, 180 min), Adsorption Efficiency Calculated with Changing Temperature Values (50 mL, 100 mg/L Nd^3+^, 0.2 g adsorbent, 5.5 pH, 180 min), and Adsorption Efficiency Calculated with the Varying Solid to Liquid Ratio (50 mL, 100 mg/L Nd, 25 °C, 5.5 pH, 180 min)

parameters			
pH	3.5	5.5	7.5
adsorption efficiency	17.69	26.80	17.24
adsorbant to liquid ratio (g/mL)	1/250	1/50	
adsorption Efficiency	26.80	76.84	
temperature (°C)	25	55	75
adsorption efficiency	26.8	31.54	60.75

#### 3.3.4 Effect of Temperature on Adsorption

To observe the effect of temperature on the adsorption process, three different experiments were carried out at 25, 55, and 75 °C for 180 min each with 50 mL of a solution containing 100 mg/L Nd^3+^ ion and 0.2 g of adsorbent, and the adsorption efficiencies are given in Table 4. Increasing the temperature from 25 to 55 °C increased the efficiency by 4.74% while increasing it to 75 °C increased it by 33.95%. In the study of Zhang et al., the effect of temperature was observed at 15, 20, 30, and 45 °C in the study of Cu^2+^ ion adsorption from wastewater with alginate-based magnetic gels; an increase in inefficiency was observed with an increase in temperature, but a striking increase was observed.^56^ In this study, increasing from 25 to 55 °C did not have a striking effect on the system, but increasing it from 55 to 75 °C showed a striking effect. The reason for this is that the *q*_e_ value was tried to be kept low by considering the deadsorption studies in this study, and the adsorption efficiency was tried to be increased significantly with the increasing ion mobility with the effect of temperature. In other literature studies where the temperature did not show a striking effect, the efficiency was greater than 65% at room temperature.^22,56,58^ It has been seen that increasing the efficiency with the increase in temperature provides convenience in deadsorption studies rather than the higher efficiency of the material at room temperature. In addition, the increase in yield with temperature increases indicates that the process is endothermic.^56^

#### 3.3.5 Effect of the Solid-to-Liquid Ratio on the Adsorption Process

To observe the effect of the solid-to-liquid ratio on the adsorption process, two different experiments were carried out for 180 min each with 50 mL of a solution containing 100 mg/L Nd^3+^ ion, 0.2, and 1 g of adsorbent material at 25 °C and the adsorption efficiencies are given in Table 4. As expected, the efficiency increased as the amount of the adsorbent increased. Efficiency is increased as more adsorbent recognizes more adsorption surface area.

#### 3.3.6 Adsorption Efficiency

**Figure 13:**
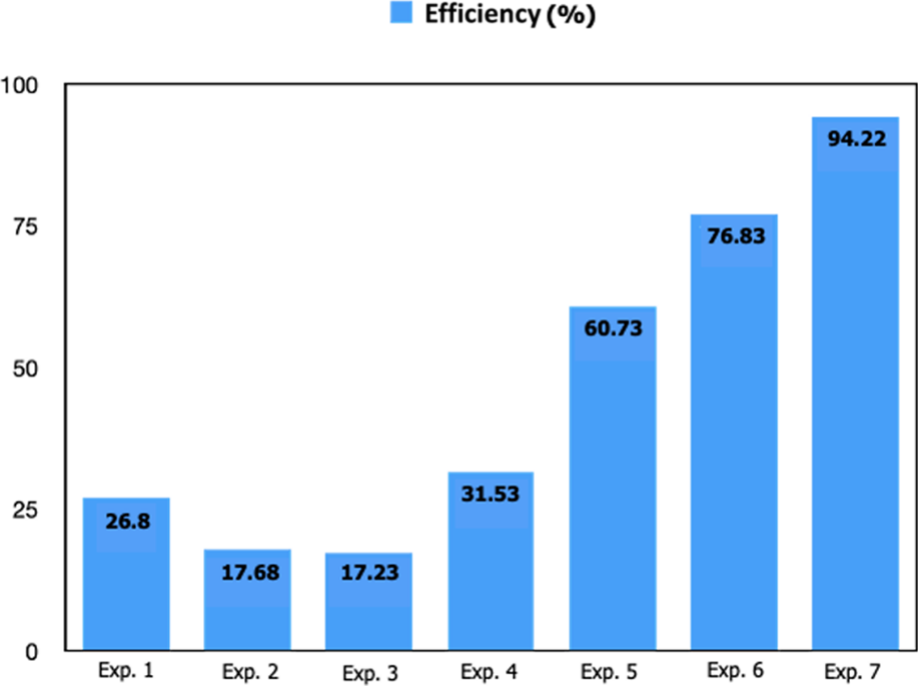
Adsorption efficiency of seven different experiments.

The adsorption efficiency of seven experiments is given in Figure 13. It was observed that the adsorption efficiency increased to 94.22% by using optimized pH, temperature, and solid/liquid parameters with 50 mL of a solution containing 100 mg/L Nd^3+^ ions. It has been understood that the adsorption efficiency can be increased not only by the chemical and physical properties of the adsorbent material but also by optimizing the adsorption parameters.

#### 3.3.7 Thermodynamic Analysis of Adsorption

Thermodynamic variables effective in the adsorption process were calculated for three different temperatures and are given in Table 5. It was made with a 50 mL solution containing 100 mg/L Nd^3+^ ion and 0.2 g of adsorbent material at 25, 55, and 75 °C temperatures. Negative Gibbs free energy was obtained at three different temperatures; the adsorption reaction occurs spontaneously, with positive enthalpy values; adsorption is endothermic, with positive entropy values, showing that the reaction is entropy-driven.^22^

**Table 5:** Effective Thermodynamic Parameters in the Adsorption Process (100 mg/L Nd^3+^, 50 mL of Solution, pH 5.5, and 0.2 g of Adsorbent)

temperature (Kelvin)	Δ*G*^0^ (kJ/mol)	Δ*H*^0^ (kJ/mol)	Δ*S*^0^ (J/mol K)
298	–1.51	0.32	1.15
328	–2.27	0.32	1.15
348	–5.92	0.32	1.15

#### 3.3.8 Effect of the Initial Concentration on Adsorbent Capacity

**Figure 14:**
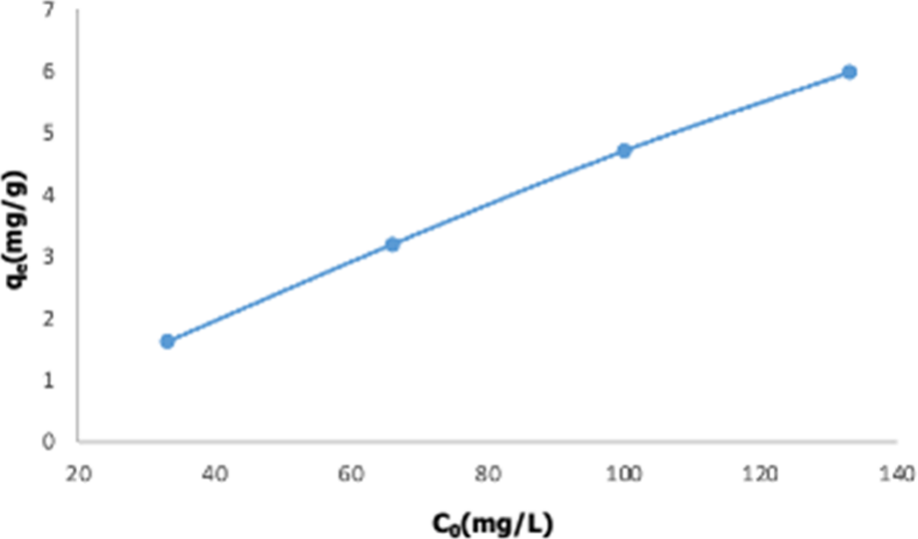
Adsorption capacity of the adsorbent changes with varying initial metal concentrations (50 mL of solution, 1 g of adsorbent, 75 °C).

The adsorbent capacity varying with the initial concentration of the Nd^3+^ ion is given in Figure 14. Four different 50 mL solutions containing 133, 100, 66, and 33 mg/L Nd^3+^ ions were prepared, and adsorption experiments were carried out for 180 min with pH 5.5, 1 g adsorbent material. It is seen that with increasing initial metal concentration, the adsorbent capacity also increases. More collisions occur between metal ions and active adsorbent surfaces in the solution, and as a result, the capacity of the adsorbent increases in proportion to the initial concentration.^21^

#### 3.3.9 Adsorption İsotherms

**Figure 15:**
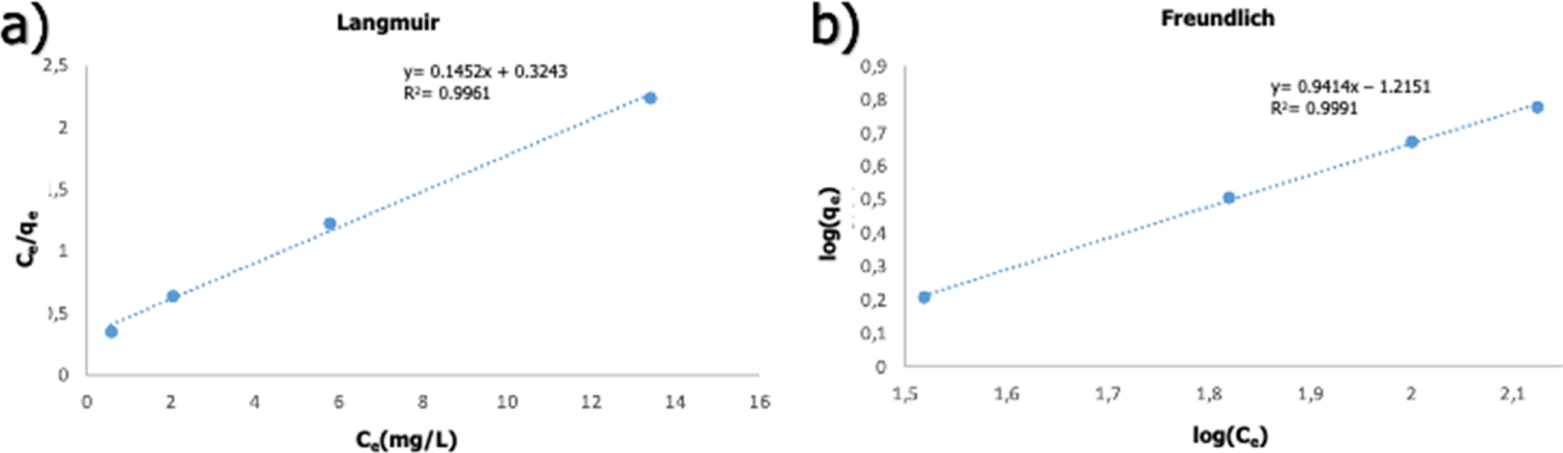
Fitting experimental data to Langmuir (a) and Freundlich (b) models.

Experimental results were fitted to Langmuir and Freundlich isotherm models to better understand the adsorption behavior of Nd^3+^ ions. *C*_e_/*Q*_e_ versus *C*_e_ is plotted for the Langmuir isotherm model; log(*q*_e_) versus log(*C*_e_) is plotted for the Freundlich model and results are given in Figure 15. The variables calculated from isotherm models are given in Table 6. When the *R*_L_ value was calculated for all concentrations, it was observed that it remained between 0 and 1, indicating a favorable adsorption process. When the Freundlich isotherm model is examined, it is seen that 1/n is less than 1, that is, the reaction is normal adsorption. When the overall compatibility of the two models is examined, the experimental data fit the Freundlich model with a value of *R*^2^ = 0.9991. Therefore, the adsorption reaction occurs heterogeneously in multiple planes and on the surface.^65^

**Table 6:** Langmuir and Freundlich Models’ Constants

Langmuir model			Freundlich model		
*Q*_m_ (mg/g)	*K*_L_ (L/mg)	*R*^2^	*k*_f_ [(mg/g)(L/g)^1/n^]	*n*_f_	*R*^2^
6.88	0.44	0.9961	0.06	1.06	0.9991

#### 3.3.10 Deadsorption Studies

**Figure 16:**
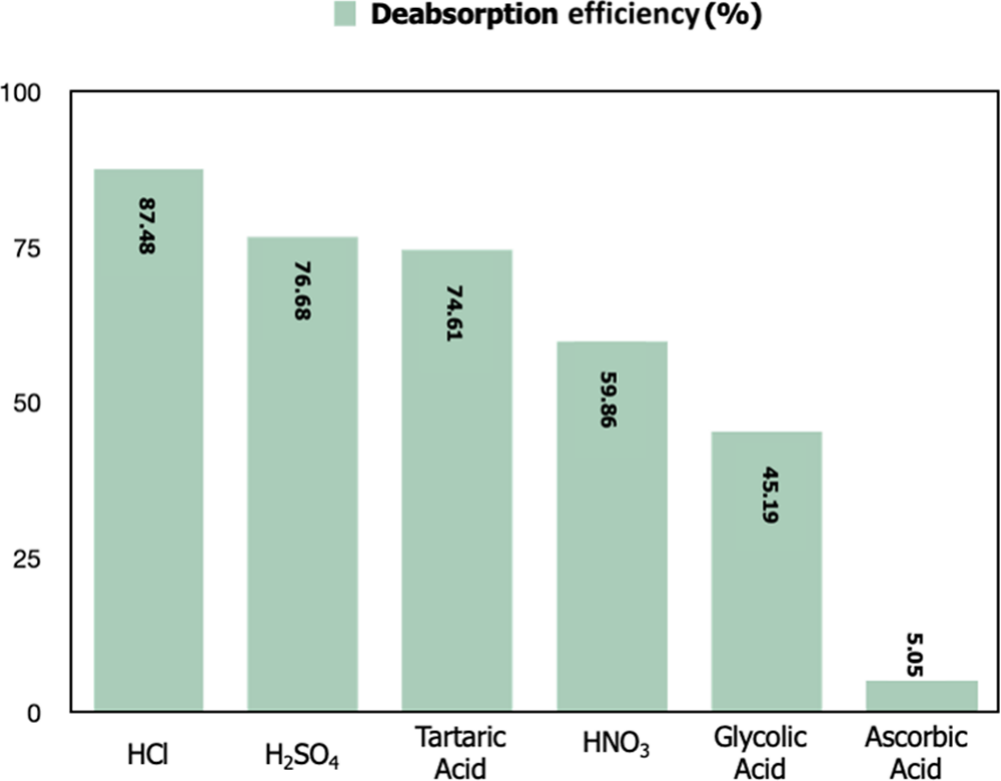
Efficiency of deabsorption studies in different acid media.

Recovery of Nd^3+^ ions by deadsorption after adsorption is important due to economic and environmental factors. After adsorbing the Nd^3+^ ions from the aqueous medium, chemical regeneration of the adsorbent material was carried out using various acids (0.1 M HCl, 0.1 M H_2_SO_4_, 0.1 M tartaric acid, 0.1 M HNO_3_, 0.1 M glycolic acid, and 0.1 M ascorbic acid). Obtained deadsorption results are given in Figure 16. While the highest efficiency was obtained with HCl (88.48%), the lowest efficiency was observed with ascorbic acid (5.05%). Compared to the deadsorption efficiencies obtained in literature studies, very high deadsorption efficiencies were obtained with metal-acid complexes.^21^ The mineral acids were at a sufficient level to disrupt the interaction between the adsorbent and metal ions. In organic acids, tartaric acid showed a high removal efficiency. The reason for the high efficiency, of tartaric acid, is the complex formation between tartaric acid and REMs.^66^ The deadsorption efficiency of other organic acids was lower than that of mineral acids. Furthermore, glycolic acid showed greater deadsorption efficiency than ascorbic acid. In addition, it was reported that glycolic acid at the same concentration showed much higher resolution efficiency than ascorbic acid in organic acid leaching studies.^67^ It has been reported that the difference in the efficiency of glycolic and ascorbic acid in leaching processes is related to pKa (acid strength).^67^ For the same reason, it was thought that the efficiencies of these acids are different.

## 4 Conclusions

In this study, magnetic gel production was carried out successfully to be used in the cleaning of heavy metals and REMs from wastewater by the magnetic separation method. Magnetic Fe particles with an oxide layer of 16 nm and an average size of 400 nm were synthesized by the polyol method. It was observed that ethylene glycol was oxidized to CO_3_^–^ during the Fe reduction reaction. The synthesized particles were added to the gels and ferromagnetic gels were produced successfully. It was determined by FT-IR and DSC analysis that the addition of Fe particles to the gels was effective on the Egg-Box structure. Then, absorption of Nd from wastewater experiments was done and it achieved 94.22% absorption efficiency. Temperature, time, pH, and solid-to-liquid ratio’s effects on the absorption process were investigated. It was observed that the adsorption efficiency was the highest at 75 °C, 5.5 pH, and 1/50 absorbent liquid ratio. Furthermore, kinetic models, isotherms, and thermodynamic constants were calculated. It has been observed that the adsorption process does not fit a single kinetic model but fits different models at different time intervals. It has been observed that the adsorption isotherm conforms to the Freundlich Isotherm model, that is, adsorption takes place from heterogeneous and multiple planes. When the thermodynamic dimensions of the process are examined, it was observed that the reaction developed spontaneously was an endothermic process and was entropy-driven. After the absorption process, deabsorption experiments were done and absorbed Nd^3+^ ions get the solution back with 87.48% efficiency with HCl acid. However, Nd^3+^ ions were recovered with more than 70% efficiency with organic acids such as tartaric acid.

## Data Availability

Since the raw/processed data are also used in an ongoing study, it is not currently possible to share them to replicate these results.

## Supporting Information Available

Crystallite size calculations of Fe-Based submicron particles produced with ethylene glycol by W–H and Modified Debye Scherrer approaches; dislocation density calculated by W–S analysis; results of SEM–EDS analysis performed without exposing the particles to washing steps; adsorption kinetics models applied for all time intervals and model constants; and pourbaix diagram of Nd^3+^ and SO^4–^ ions (PDF)
