# Supplementary material for: Nd Recovery from Wastewater with Magnetic Calcium Alginate ((1,4)-β-d-Mannuronic Acid and α-L-Guluronic Acid) Hydrogels
Source: ACS Omega. 2023 May 3;8(19):16762–78. doi: 10.1021/acsomega.2c08221 (PMC10193390; doi:10.1021/acsomega.2c08221)
Supplement: Supplementary file 1 — ao2c08221_si_001.pdf [file ao2c08221_si_001.pdf]

Supplementary Information

Nd Recovery from Wastewater with Magnetic Calcium Alginate ((1,4)- β -d-Mannuronic Acid and α -L-Guluronic Acid) Hydrogels

Emircan Uysal [a], Elif Emil-Kaya [a,b], Duygu Yesiltepe-Ozcelik [a], Sebahattin Gurmen [a]*

a) Department of Metallurgical and Materials Engineering, Istanbul Technical University, 34469, Istanbul, Türkiye

b) IME Process Metallurgy and Metal Recycling, RWTH Aachen University, DE 52062, Nodrhein-Westfalen, Aachen, Germany

[*]Correspondence to;

Sebahattin Gürmen, Department of Metallurgical and Materials Engineering, Istanbul Technical University, Istanbul, Türkiye, e-mail; gurmen@itu.edu.tr

S1. W-H and. MDS analysis of Fe-Based Particles

Firstly, three peaks are assigned to (101), (200), and (211) planes selected afterward peak widths were determined by X'Pert High Score Plus software. X-ray diffraction peak broadening is constituted by strain and crystallite size. Strain-induced peak broadening is evaluated as crystal defects and given as ε = βs/tanθ.

The X-ray diffraction peak broadening was calculated by subtracting instrumental peak broadening from the measured peak broadening as given in eq S1.

$$\beta_{hkl} = [\beta^2_{measured} - \beta^2_{instrumental}]^{\frac{1}{2}} \tag{S1}$$

The contribution of crystallite size and lattice strain to total X-ray peak broadening is given in eq S2.

$$\beta_{hkl} = \beta_{crystallite} + \beta_{strain} \tag{S2}$$

Crystal distortion can be calculated with eq S3.

$$\varepsilon \approx \beta_s/\tan\theta \tag{S3}$$

Eqs S4 and S5 are obtained by using Debye–Scherrer equation and the abovementioned equations.

$$\beta_{hkl} = \left(\frac{k\lambda}{Dcos\theta}\right) + (4\varepsilon\tan\theta) \tag{S4}$$

$$\beta_{hkl} cos\theta = \left(\frac{k\lambda}{D}\right) + (4\varepsilon \sin\theta) \qquad (S5)$$

whereas β_{hkl}, θ, k, and λ state peak broadening, diffraction angle, shape factor (In this case: 0.84), and wavelength of $CuK\alpha$ radiation (λ= 0,154184 nm), respectively. Moreover, D states the average crystallite size and ε is the average lattice strain [1–3].

The graph of (βcos θ) versus (4sinθ) for the synthesized Fe particles was plotted to calculate crystallite size and lattice strain. Afterward, the values were fitted with a linear equation. The strain is equal to the line's slope. Crystallite size is represented by the location of the line's y-axis intersection. Fig. 1b represents the W-H analysis of the synthesized Fe particles.

The crystallite size was calculated as 34 nm by W-H analysis based on a uniform deformation model. MDS analysis was performed to compare the W-H analysis. Figure 1c shows the plot of the MDS analysis.

Crystallite sizes of the synthesized Fe particles were approximately 21 nm. As the lattice strain was not taken into account, as expected, the crystallite size values determined by the MDS approach differ from those determined by the W-H method. The MDS approach calculates smaller crystallite sizes than the W-H method because the lattice contains tensile stress. Similar findings were reported elsewhere [3–7].

Williamson-Smallman (W-S) analysis was employed to determine average dislocation density by using crystallite sizes obtained by Williamson-Hall analysis. Eq S6 gives the W-S equation.

$$\delta = \left(\frac{1}{D^2}\right) \qquad (S6)$$

The average dislocation density of the synthesized Fe particles was calculated as 8.65×10^{-4} δ.

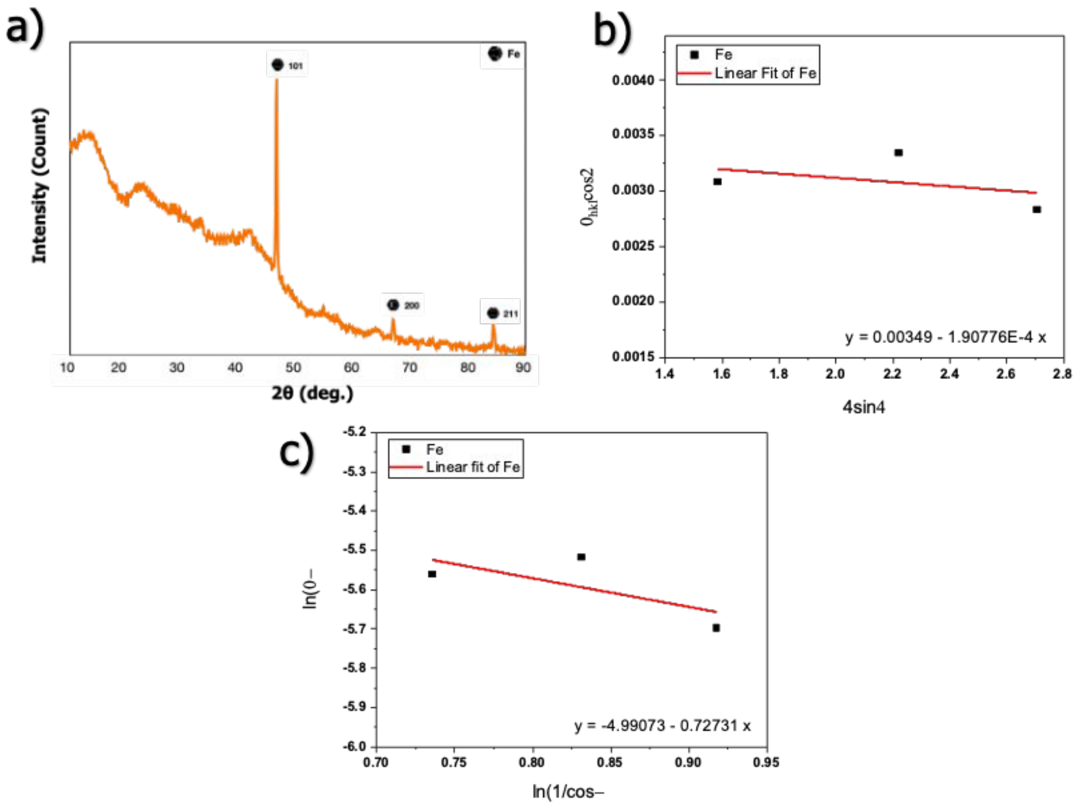

Figure S1. XRD pattern of synthesized Fe particles (a), W-H analysis of the synthesized Fe particles (b), and MDS analysis of the synthesized Fe particles (c)

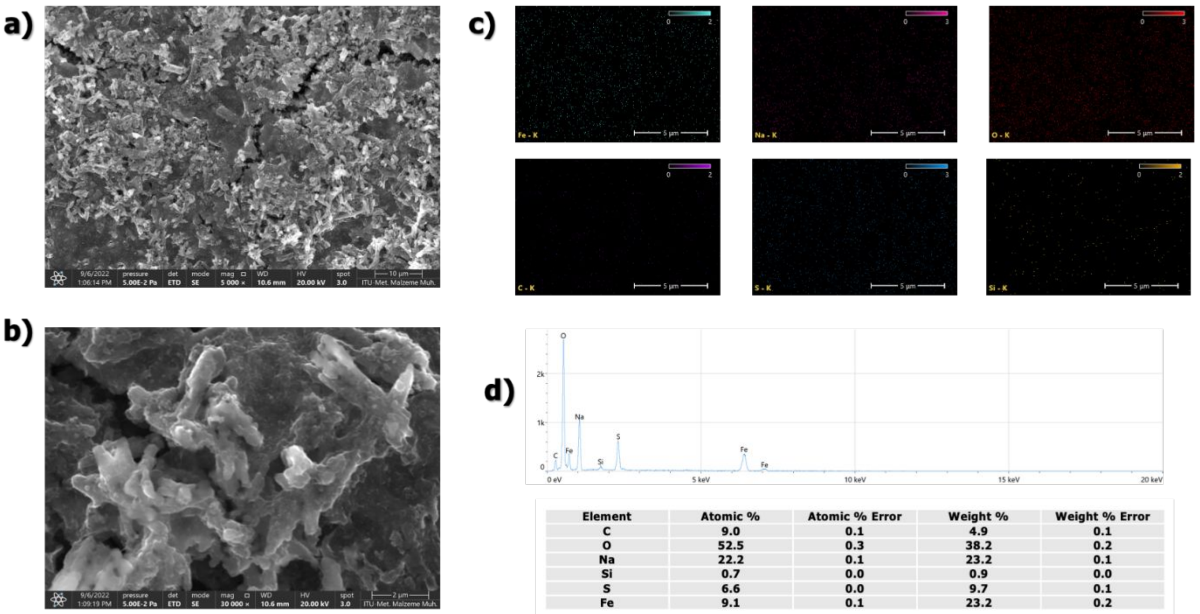

Element	Atomic %	Atomic % Error	Weight %	Weight % Error
C	9.0	0.1	4.9	0.1
O	52.5	0.3	38.2	0.2
Na	22.2	0.1	23.2	0.1
Si	0.7	0.0	0.9	0.0
S	6.6	0.0	9.7	0.1
Fe	9.1	0.1	23.2	0.2

Figure S2. SEM/EDS analysis of unwashed particles;(a) x5000 magnification, (b) x30000 magnification, (c) EDS maps, (d) EDS data and elemental compositions

Table S1. Calculated variables from the absorption data of the pseudo-first-order, pseudo-first-order, Weber-Morris, and Elovich equations

Pseudo-First-Order			Pseudo-Second-Order		
q_e calculated (mg/g)	K_1(min.$^{-1}$)	R^2	q_e calculated (mg/g)	K_2(g/mg.min.)	R^2
10.0646	0.0243	0.5755	7.8308	0.0027	0.9705
**Weber-Morris**			**Elovich**		
K_d (mg/g.min.$^{-1/2}$)	R^2		α (mg/t.g)	β (g/mg)	R^2
0.4201	0.9218		10.2709	1.7892	0.9574

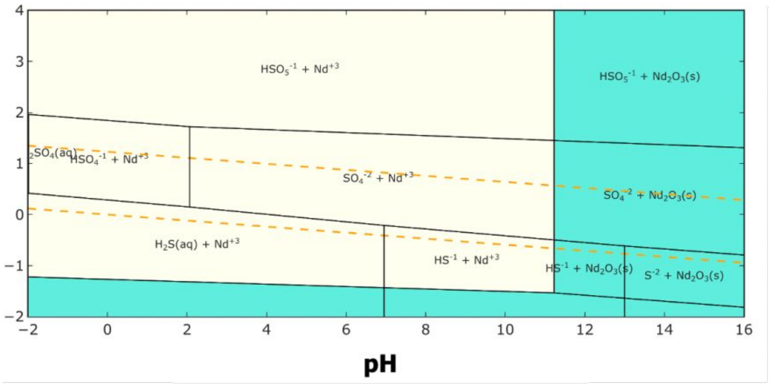

Figure S3. pourbaix diagram of Nd^{3+} and SO^{4-} ions [8–10]

References

(1) Dorset, D. L. X-Ray Diffraction: A Practical Approach. *Microsc Microanal* **1998**, *4* (5), 513–515. https://doi.org/10.1017/S143192769800049X.

(2) *Fundamentals of Powder Diffraction and Structural Characterization of Materials*; Springer US: Boston, MA, 2009. https://doi.org/10.1007/978-0-387-09579-0.

(3) Khorsand Zak, A.; Abd. Majid, W. H.; Abrishami, M. E.; Yousefi, R. X-Ray Analysis of ZnO Nanoparticles by Williamson–Hall and Size–Strain Plot Methods. *Solid State Sciences* **2011**, *13* (1), 251–256. https://doi.org/10.1016/j.solidstatesciences.2010.11.024.

(4) Emil, E.; Gürmen, S. Estimation of Yttrium Oxide Microstructural Parameters Using the Williamson–Hall Analysis. *Materials Science and Technology* **2018**, *34* (13), 1549–1557. https://doi.org/10.1080/02670836.2018.1490857.

(5) Kaya, E. E.; Gürmen, S. A Straightforward Approach for the Synthesis of Nanostructured Y2O3 Particles: Synthesis, Morphology, Microstructure and Crystal Imperfection. *Physica E: Low-dimensional Systems and Nanostructures* **2020**, *115*, 113668. https://doi.org/10.1016/j.physe.2019.113668.

(6) Uvarov, V.; Popov, I. Metrological Characterization of X-Ray Diffraction Methods for Determination of Crystallite Size in Nano-Scale Materials. *Materials Characterization* **2007**, *58* (10), 883–891. https://doi.org/10.1016/j.matchar.2006.09.002.

(7) Akl, A. A.; Mahmoud, S. A.; AL-Shomar, S. M.; Hassanien, A. S. Improving Microstructural Properties and Minimizing Crystal Imperfections of Nanocrystalline Cu 2 O Thin Films of Different Solution Molarities for Solar Cell Applications. *Materials Science in Semiconductor Processing* **2018**, *74*, 183–192. https://doi.org/10.1016/j.mssp.2017.10.007.

(8) Singh, A. K.; Zhou, L.; Shinde, A.; Suram, S. K.; Montoya, J. H.; Winston, D.; Gregoire, J. M.; Persson, K. A. Electrochemical Stability of Metastable Materials. *Chem. Mater.* **2017**, *29* (23), 10159–10167. https://doi.org/10.1021/acs.chemmater.7b03980.

(9) Patel, A. M.; Nørskov, J. K.; Persson, K. A.; Montoya, J. H. Efficient Pourbaix Diagrams of Many-Element Compounds. *Phys. Chem. Chem. Phys.* **2019**, *21* (45), 25323–25327. https://doi.org/10.1039/C9CP04799A.

(10) Persson, K. A.; Waldwick, B.; Lazic, P.; Ceder, G. Prediction of Solid-Aqueous Equilibria: Scheme to Combine First-Principles Calculations of Solids with Experimental Aqueous States. *Phys. Rev. B* **2012**, *85* (23), 235438. https://doi.org/10.1103/PhysRevB.85.235438.
